# Revisiting Bill Lands’ Hypotheses: HUFA Balance, Immuno-Metabolic Regulation, and Conflicting Clinical Evidence

**DOI:** 10.3390/nu18040626

**Published:** 2026-02-13

**Authors:** Ulrich Suchner

**Affiliations:** Department of Anesthesiology and Surgical Intensive Care Medicine, Klinikum Darmstadt, 64283 Darmstadt, Germany; carpediemusu@outlook.de

**Keywords:** omega-6, omega-3, linoleic acid, seed oil, health, disease

## Abstract

The optimal dietary balance between *n*-6 and *n*-3 polyunsaturated fatty acids (PUFAs), the safe upper intake of *n*-6 PUFAs—particularly linoleic acid—and the physiological consequences of their metabolic competition remain unresolved in the context of the Western diet. Since the 1980s, Bill Lands and colleagues have argued that high *n*-6 PUFA intake can shift the balance of *n*-3-derived pathways and eicosanoid signaling, potentially influencing processes relevant to non-communicable diseases. Across human populations, the proportion of *n*-6 in tissue HUFA spans a broad range—from roughly 20% in traditional dietary patterns to nearly 80% in typical Western diets—illustrating the predictable impact of dietary precursor supply on HUFA composition. Despite its potential public health implications, this hypothesis has received limited systematic attention. In this narrative review, we synthesize key aspects of Lands’ work, evaluate supportive and contradictory evidence, and highlight mechanistic insights into lipid competition and inflammatory regulation. We conclude that these unresolved but testable hypotheses warrant renewed investigation, as their corroboration could reshape dietary guidelines and strategies for chronic disease prevention.

## 1. Introduction—Why This Article Was Written

Bill Lands and his co-workers have pursued many research interests and developed many important scientific contributions in the biochemistry of lipids. Recently, new appreciation was granted for an “old pathway”—the so-called “Lands Cycle”—that “moves science into new arenas in health and disease” [1]. Further to this, Bill Lands work on the contrasting influences of *n*-6 and *n*-3 polyunsaturated fatty acids (PUFAs) in the so-called “Western diet” is of outstanding importance and may even surpass the formerly mentioned contribution. Lands’ overriding hypothesis on the latter topic can be summarized pointedly by stating that there is a “*n*-6 PUFA-related nutritional healthcare problem” of global importance [2]. That healthcare problem likely originates in humankind’s ability to produce plant seed oils containing large amounts of the *n*-6 PUFA linoleic acid, such as soybean, corn and sunflower oils, to industrialize the production of foods that contain such oils, and to market these derived products successfully. But what is the human health impact of this? Despite the progress in pathophysiological understanding and therapeutic procedures related to the so-called “chronic lifestyle diseases”, the ultimate understanding of all of the relevant causal relationships for their development is still missing. According to Lands, however, these diseases result from a causal chain of a global nutrition issue that is driven by the steadily growing (over)supply of dietary *n*-6 PUFAs (especially linoleic acid) over the last 100 years [2,3]. Whereas we share the appraisal of the relevance of that hypothesis by Lands—although it certainly awaits further verification—we also share his perception that there is a neglect of the available line of reasoning by the scientific as well as medical community and by political, health care as well as industrial stakeholders. To date, the therapeutic focus has tended to be on symptomatic treatment approaches for non-communicable diseases that have already appeared, with prevention being largely disregarded. This continues to happen, even though the successful “preventive approach” to a health problem is always preferable to its therapeutic remedy (“prevention is better than treatment”). However, it seems that the “symptomatic treatment approach” appears to be appreciated as more elegant and fascinating from a scientific or technical point of view. Not least, the assumption cannot be ruled out that the symptomatic treatment approach of diseases, once they emerge, is much more beneficial for healthcare providers, manufacturers, and suppliers. Against this background, this article aims to recall Bill Lands’ hypotheses on the overavailability of *n*-6 PUFAs and the related pathologies, review the supporting and opposing data, and assess their significance along with their further scientific verification.

## 2. The Essence of Lands’ Hypotheses

Lands introduced the concept of HUFA-based metrics—most notably the percentage of *n*-6 in HUFA—to quantify the relative contributions of 20- and 22-carbon highly unsaturated fatty acids (HUFAs) derived from *n*-3 and *n*-6 polyunsaturated fatty acids (PUFAs), and to distinguish these long-chain HUFAs from their 18-carbon PUFA precursors. The focus on HUFAs is because they are highly bioactive in comparison to their 18-carbon precursors, particularly as substrates for classical eicosanoid pathways [3,4,5,6,7]. At the same time, recent work has shown that 18-carbon PUFAs such as linoleic acid and α-linolenic acid can also give rise to biologically active metabolites with regulatory functions [8]. Thus, the emphasis on HUFAs reflects their central role in well-characterized mediator pathways, while not implying that their 18-carbon precursors are biologically inert.

In this section, we outline and discuss the key hypotheses formulated by Lands regarding PUFA metabolism, HUFA dynamics, and their implications for health and disease. These hypotheses are summarized in Table 1, which presents them in a logical progression from biochemical mechanism to quantitative modeling, pathophysiological consequences, nutritional intervention, and public-health relevance.

### 2.1. The Dietary Mixture of Polyunsaturated Fatty Acids (PUFAs) Determines Cellular Fatty Acid Profiles and Thereby Shapes the Non-Energetic Biological Actions of These Lipids

**Figure 1:**
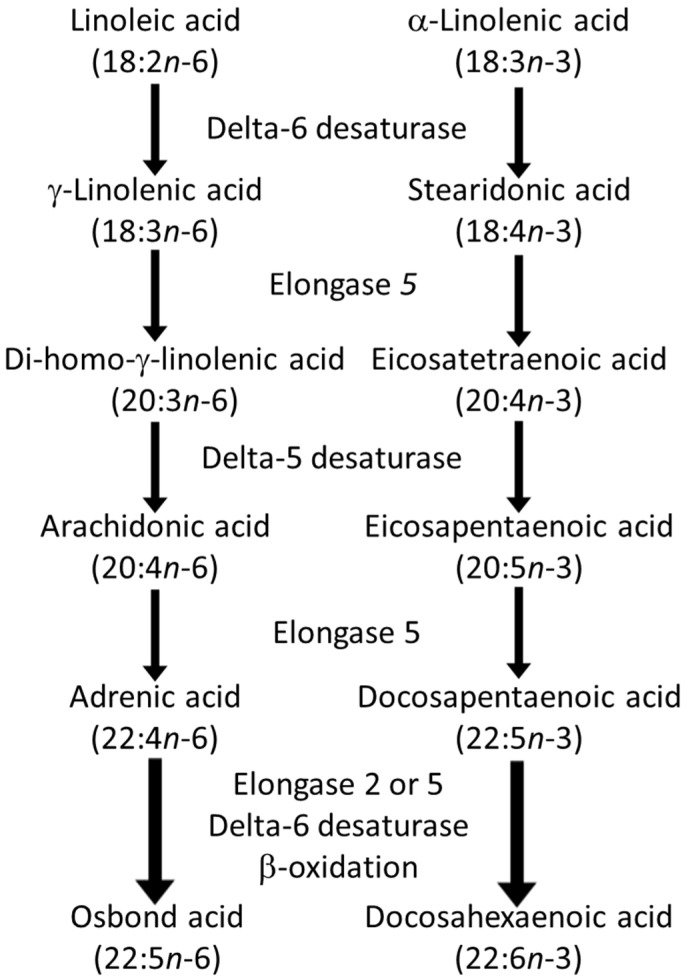
Biochemical pathway for conversion of linoleic and α-linolenic acids to longer chain, more unsaturated fatty acids. Note that the two pathways share the same enzymes creating direct competition between *n*-6 and *n*-3 fatty acid families for metabolism.

PUFAs are bioactive, influencing physiology and determining risk and severity of disease [9]. The relative dietary supplies of competing *n*-6 and *n*-3 PUFAs, including preformed HUFAs, determine the tissue balance of 20- and 22-carbon *n*-6 and *n*-3 HUFAs, which accumulate competitively at the 2-position of tissue phospholipids [10]. There is metabolic competition between the 18-carbon PUFAs for conversion to HUFAs (Figure 1), which is determined by the relative supply of linoleic acid (LA; 18:2*n*-6) and α-linolenic acid (ALA; 18:3*n*-3).

**Figure 2:**
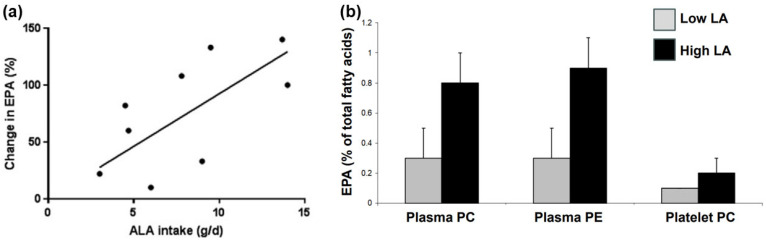
Effect of increasing intake of ALA (**a**) and decreasing intake of LA (**b**) on EPA status. (**a**) Change in plasma phospholipid EPA as a result of increased ALA intake using data from eight different human studies. Reprinted from [16]: Progress in Lipid Research, Vol 64, E.J. Baker et al., Metabolism and functional effects of plant-derived omega-3 fatty acids in humans, Pages 30–56, Copyright (2016), with permission from Elsevier. (**b**) EPA (% of total fatty acids) in plasma phosphatidylcholine (PC), plasma phosphatidylethanolamine (PE) and platelet PC in healthy young adults after 18 days of consuming foods providing a low LA intake (19% of fatty acids) or a high LA intake (44% of total fatty acids) but the same intake of ALA (6.5% of fatty acids). Data are taken from Chen et al. [17].

**Figure 3:**
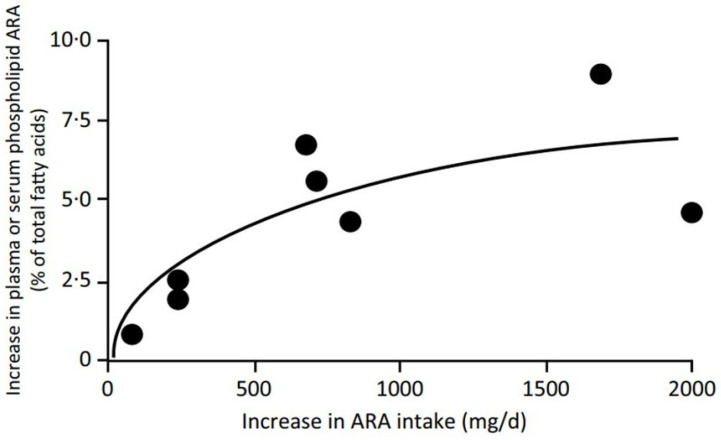
The relationship between supplemental arachidonic acid (ARA) intake (mg/d) and increment in ARA in serum or plasma phospholipids (as percentage of total fatty acids). The figure is taken from Calder et al. [18] (Open Access: CC BY 4.0) and shows mean data from seven human trials that involved eight different supplemental intakes of ARA.

**Figure 4:**
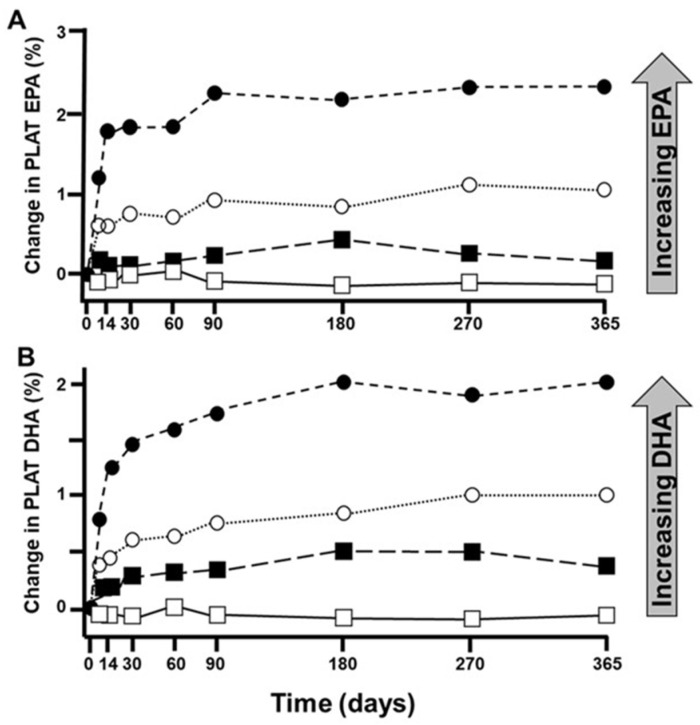
Time course of changes in eicosapentaenoic acid (EPA; (**A**)) and docosahexaenoic acid (DHA; (**B**)) content of human platelets (PLAT) in participants consuming placebo oil or one of three doses of EPA+DHA. Healthy participants supplemented their diet with capsules providing 0 (solid line), 3.27, 6.54 or 13.08 (dotted lines) g EPA + DHA per week for a period of 12 months; the ratio of EPA to DHA was 1:1.1. Platelets were isolated from blood at 0, 1, 2, 4, 8, 12, 24, 36 and 52 weeks and the fatty acid composition determined by gas chromatography. Data are means from at least 30 participants per group. Reprinted from [19]: The American Journal of Clinical Nutrition, Vol 99, L.M. Browning et al., Incorporation of eicosapentaenoic and docosahexaenoic acids into lipid pools when given as supplements providing doses equivalent to typical intakes of oily fish, Pages 748–758, Copyright (2012), with permission from The American Society for Nutrition.

The pathway involves the sequential activities of the ∆6 fatty acid desaturase, elongase 5 and ∆5 fatty acid desaturase enzymes, which convert LA to ARA and ALA to EPA (Figure 1). At the high intakes of LA present in many diets, including the “Western diet”, the pathway is saturated with LA such that differences in LA intake are not related to differences in ARA content of blood or tissues [11,12]. However, it is important to note that the ∆6 fatty acid desaturase enzyme (encoded by the gene *FADS2*) prefers the *n*-3 PUFA substrate (i.e., ALA) over the *n*-6 PUFA substrate (i.e., LA) [13,14]. However, a high LA intake, such as that characterizing Western diets, suppresses the desaturation of ALA [15], i.e., the much higher dietary intake of LA than of ALA limits the conversion of dietary ALA to its metabolites further downstream (e.g., EPA and DHA). That this competition is a reality in the human setting is demonstrated by trials showing that increasing intake of ALA results in higher EPA in blood and blood cells as reviewed elsewhere [16] (Figure 2a) and that decreasing LA intake also results in higher EPA in blood and blood cells [17] (Figure 2b). Higher intake of ARA results in higher levels of ARA in blood, cells and tissues as reviewed elsewhere [18] (Figure 3), partly at the expense of EPA and DHA, while higher intake of EPA and DHA results in higher levels of EPA and DHA in blood, cells and tissues [19] (Figure 4) partly at the expense of ARA [20]. Hence, dietary intake of LA, ALA, ARA, EPA and DHA all influence the level of *n*-6 and *n*-3 HUFAs in blood, cells and tissues.

### 2.2. n-6 and n-3 Highly Unsaturated Fatty Acids (HUFAs) Influence Each Other Metabolically, Differ in Their Biochemical Efficacy, and Give Rise to Distinct Organ- and System-Level Functions

**Figure 5:**
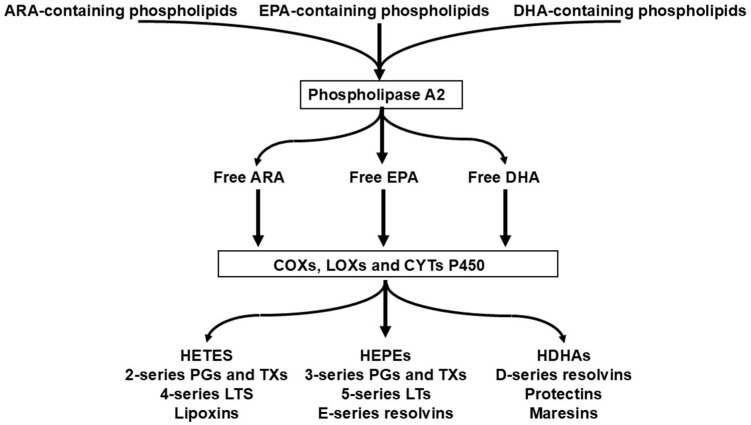
Overview of the conversion of highly unsaturated fatty acids to bioactive lipid mediators highlighting the competition that exists for the various enzymes involved. Note: Arachidonic acid and docosahexaenoic acid are preferentially released from phosphoglycerides by different types of phospholipase A2, as reviewed by Murawaska et al. [21]. COX, cyclooxygenase; CYT, cytochrome; HDHA, hydroxydocosahexaenoic acid; HEPE, hydroxyeicosapentaenoic acid; HETE, hydroxyeicosatetraenoic acid; LOX, lipoxygenase; LT, leukotriene; PG, prostaglandin; TX, thromboxane.

As indicated above, there is direct metabolic competition between *n*-6 and *n*-3 HUFAs for incorporation into complex lipids such as the phospholipids that make up cell membranes. Subsequently, there is competition for the enzymes that release HUFAs from phospholipids (e.g., phospholipase A2) and for the enzymes that generate bioactive oxylipin metabolites such as prostaglandins, thromboxanes and leukotrienes (Figure 5).

The actions of the lipid mediators produced from ARA often differ in intensity from those derived from EPA and DHA. These differences can lead to distinct patho-physiological consequences depending on the proportional distribution of *n*-6 and *n*-3 HUFAs in cell membranes, that is, the relative % *n*-6 and % *n*-3 within the HUFA pool (for terminology, see Section 2.4). The percentage of *n*-6 in tissue HUFAs directly relates to the severity of morbid conditions caused by excessive *n*-6 HUFA-related actions. Notably, in the absence of *n*-3 PUFAs, dietary LA has a very narrow therapeutic window—even if provided below 1 percent of food energy. But this window can be widened by either the supply of *n*-3 PUFAs or by the reduction in n-6 PUFA uptake [3]. While the first option has been receiving increasing attention for years, the second option of dietary reduction in *n*-6 PUFAs has been largely neglected. Lands has drawn attention to this.

Beyond the supply of the metabolic substrates, LA and ALA, variations in the operation of the conversion pathway shown in Figure 1 result from genetic variations (single-nucleotide polymorphisms) in the *FADS1* and *FADS2* genes [22]. Recently, Brenna et al. [23] drew attention to functional genetic variants in *FADS1* that partly define the levels of circulating ARA and may predispose to disease. In particular, it is predicted that “fast” desaturators (insertion allele at rs66698963 of *FADS1*; the major haplotype in Europeans) are predisposed to a higher risk of pathological responses to SARS-CoV-2, for example [24]. There is also an interaction between the fast desaturator genotype and response to *n*-3 HUFA intervention: participants with the fast Δ5-desaturation allele had a 50% reduction in colorectal polyps when treated with 2 g/d of EPA for a year [25], when the entire cohort was unresponsive to the intervention [26], suggesting that *n*-3 HUFA intervention may be more effective in those genetically predisposed to higher *n*-6 HUFA levels.

Epigenetic regulation and gene–diet interactions likewise modulate desaturase activity. These developments lie beyond the historical scope of Lands’ hypotheses but are fully compatible with—and in some respects further support—the mechanistic principles outlined here; they are therefore not considered in detail.

### 2.3. The Competition of n-6 and n-3 HUFAs for Shared Metabolic Enzymes (COX, LOX, CYP) Is the Primary Determinant of Downstream Lipid Mediator Profiles

The metabolic fates of *n*-6 and *n*-3 highly unsaturated fatty acids (HUFAs) converge on a common set of oxygenase and monooxygenase enzymes that initiate the biosynthesis of eicosanoids and related lipid mediators. Once liberated from membrane phospholipids by phospholipase A_2_, arachidonic acid (ARA), eicosapentaenoic acid (EPA), and docosahexaenoic acid (DHA) enter a shared catalytic landscape in which cyclooxygenases (COX-1/2), lipoxygenases (5-, 12-, and 15-LOX), and cytochrome P450 epoxygenases and hydroxylases act largely as substrate-driven processors rather than as selective gatekeepers. These enzymes exhibit only modest intrinsic substrate preferences; instead, they oxygenate whichever HUFA species is most available at the active site at the moment of catalysis. This biochemical architecture creates a direct competitive interaction among HUFAs, making their relative abundance—rather than their absolute concentrations—the dominant determinant of the mediator spectrum produced.

This competition has profound consequences for the qualitative and quantitative pattern of lipid mediators. When ARA constitutes the majority of the HUFA pool, COX enzymes predominantly generate series-2 prostaglandins and thromboxanes, LOX enzymes produce series-4 leukotrienes and ARA-derived hydroxyeicosatetraenoic acids (HETEs), and CYP enzymes yield ARA-derived epoxyeicosatrienoic acids (EETs). These mediators typically display higher potency in promoting vasoconstriction, platelet aggregation, leukocyte recruitment, and other components of the acute inflammatory response. In contrast, when EPA and DHA occupy a larger fraction of the HUFA pool, the same enzymatic pathways shift toward producing series-3 prostanoids, series-5 leukotrienes, EPA- and DHA-derived epoxides and hydroxylated species, and—via downstream enzymatic cascades—specialized pro-resolving mediators (SPMs) such as resolvins, protectins, and maresins. These mediators generally exhibit reduced inflammatory intensity or actively promote the resolution phase of inflammation.

Importantly, this competitive mechanism operates continuously and systemically. Because membrane HUFA composition reflects long-term dietary patterns, the relative availability of *n*-6 and *n*-3 substrates effectively “pre-sets” the inflammatory tone of tissues by biasing the mediator repertoire that can be produced in response to physiological stimuli. In Lands’ framework, this substrate-level competition is the biochemical foundation of the HUFA balance: the proportion of *n*-6 versus *n*-3 HUFAs esterified in membrane phospholipids that determines the direction and magnitude of mediator production. The HUFA balance thus provides a mechanistic bridge between dietary intake, membrane biochemistry, enzymatic competition, and the emergent pattern of inflammatory and resolution-phase signaling.

By situating lipid mediator biosynthesis within this competitive enzymatic context, the HUFA balance concept clarifies why small shifts in membrane HUFA composition can produce disproportionately large changes in downstream signaling. It also explains why interventions that alter the *n*-6/*n*-3 HUFA ratio—whether through diet, supplementation, or metabolic modulation—can reshape inflammatory trajectories across diverse physiological and pathophysiological settings. In this sense, the competition for COX, LOX, and CYP enzymes is not merely a biochemical detail but the central organizing principle that links HUFA biology to health and disease.

### 2.4. The Quantitative Relationship Between Dietary PUFA Intake and HUFA Composition Is Predictable and Can Be Modelled with High Accuracy, Enabling Mechanistic Forecasting of Biological Outcomes

The proportional distribution of *n*-6 and *n*-3 highly unsaturated fatty acids (HUFAs) in tissue phospholipids is not a stochastic outcome of diet but follows a quantifiable, saturable, and highly reproducible relationship with dietary PUFA intake. Across species, tissues, and experimental designs, the incorporation of linoleic acid (LA)-derived and α-linolenic acid (ALA)-derived HUFAs into membrane phospholipids reflects a competitive, enzyme-limited process governed by substrate availability, elongation–desaturation kinetics, and the finite capacity of phospholipid acyltransferases. Because these processes operate under well-defined biochemical constraints, the resulting HUFA composition can be predicted with striking accuracy from dietary inputs.

Within this framework, Lands distinguishes three complementary quantitative descriptors. The *n*-6 PUFA balance refers to the dietary input ratio of *n*-6 to *n*-3 PUFA precursors that enter the competitive biosynthetic pathways.

The % *n*-6 in HUFA is a modelled, calculable quantity representing the predicted proportion of *n*-6 HUFAs in membrane phospholipids based on this dietary input and the competitive kinetics of HUFA incorporation. Thus, % *n*-6 in HUFA refers to the percentage of *n*-6-derived HUFAs within the total HUFA pool. This measure is identical to what is sometimes termed the “*n*-6 HUFA fraction”. Moreover, in the Lands literature, “HUFA balance” is employed as a general descriptor of the tissue HUFA distribution; in the present manuscript, this also corresponds to the more explicit metric % *n*-6 in HUFA.

In contrast, the *n*-6 HUFA Score is an empirically measured endpoint, obtained from tissue or blood lipid analyses, that reflects the actual, integrated HUFA composition of membranes at a given time. Together, these three quantities form a coherent system: dietary *n*-6 PUFA balance drives the predicted % *n*-6 in HUFA, and the measured *n*-6 HUFA Score provides the empirical validation of this mechanistic relationship. All these definitions are used consistently throughout this manuscript to distinguish dietary inputs, model-derived predictions, and empirically measured HUFA endpoints.

Lands and colleagues formalized the predictability through empirical and mechanistic models that relate dietary PUFA proportions to the fraction of *n*-6 and *n*-3 HUFAs in membranes. These models capture the competitive dynamics of HUFA biosynthesis and esterification, showing that the membrane HUFA profile is determined primarily by the relative dietary supply of *n*-6 versus *n*-3 precursors rather than by their absolute amounts. As dietary *n*-6 intake increases, the *n*-6 HUFA fraction rises in a sigmoidal fashion, approaching an asymptote as enzymatic pathways become saturated. Conversely, increasing dietary *n*-3 intake shifts the HUFA pool toward EPA and DHA, displacing arachidonic acid (ARA) through the same competitive mechanisms. The resulting dose–response curves are smooth, continuous, and highly predictable, enabling quantitative forecasting of membrane HUFA composition across a wide range of dietary patterns.

This predictability has profound mechanistic implications. Because the major eicosanoid-generating enzymes act on available HUFAs in proportion to their relative abundance, the membrane HUFA profile effectively presets the lipid-mediator repertoire that can be generated in response to physiological stimuli. Thus, models that accurately predict HUFA composition also enable forecasting of downstream biological outcomes, including the balance of pro-inflammatory versus pro-resolving mediators, the intensity of acute inflammatory responses, and the resolution dynamics that follow. In this sense, the quantitative relationship between dietary PUFA intake and HUFA composition provides a mechanistic bridge between nutrition, membrane biochemistry, enzymatic competition, and physiological function.

Factors such as inter-individual variation in desaturase activity, phospholipid turnover, or enzyme saturation thresholds may influence the quantitative performance of HUFA-prediction models, but these considerations extend beyond the scope of this review and do not alter the mechanistic principles underlying Lands’ framework.

By integrating dietary inputs with biochemical constraints, HUFA modelling transforms nutritional PUFA research from a descriptive field into a predictive science. It allows biological outcomes to be anticipated from first principles, clarifies why small dietary shifts can produce large changes in inflammatory tone, and provides a mechanistic foundation for interpreting interventional studies. Within Lands’ framework, this quantitative predictability is not merely a statistical convenience but a central feature of HUFA biology—one that enables mechanistic forecasting of health trajectories shaped by the HUFA balance.

### 2.5. The Long-Standing Neglect of Dietary PUFA Imbalance May Contribute to the Continued Rise in Non-Communicable Diseases

For several decades, nutritional science has paid limited attention to the competitive dynamics between dietary *n*-6 and *n*-3 PUFAs and their downstream incorporation into highly unsaturated fatty acids (HUFAs). According to Lands, this lack of interest has delayed recognition of the dietary *n*-6/*n*-3 imbalance that characterizes modern Western eating patterns. Because the relative dietary supply of these precursors influences the HUFA balance in membranes, a sustained predominance of *n*-6 PUFA intake is biochemically expected to shift tissue HUFA composition toward arachidonic-acid-rich profiles, thereby increasing the potential for producing more potent pro-inflammatory lipid mediators. In Lands’ framework, such shifts represent a mechanistic displacement of physiological homeostasis toward a state more permissive of pathophysiological processes [5].

Over the same period, non-communicable diseases (NCDs) such as cardiovascular disease, type 2 diabetes, non-alcoholic fatty liver disease, and chronic inflammatory disorders have risen steadily. Many of these conditions share a background of chronic, low-grade inflammation and impaired resolution. Although epidemiological findings remain mixed—particularly for cardiovascular outcomes—the possibility that long-standing neglect of dietary PUFA balance has contributed to these trends remains plausible at the mechanistic level. Observational studies have reported associations between higher *n*-6-dominant HUFA profiles and markers of inflammatory or metabolic dysregulation, but these associations do not establish causality. Rather, they highlight a modifiable biochemical context in which inflammatory tone may be more easily perturbed.

Within this perspective, the historical oversight of PUFA balance would not merely appear as a nutritional detail but might be regarded as a missed opportunity to integrate mechanistic insights into preventive strategies. While current evidence does not demonstrate that dietary PUFA imbalance causes the rise in NCDs, Lands’ framework underscores how a chronically *n*-6-skewed HUFA pool could theoretically align with broader epidemiological patterns by shaping the biochemical environment in which metabolic, vascular, and immune dysfunction emerge. Dietary PUFA imbalance would thus remain a potentially important—yet likely historically underappreciated—determinant that might warrant further investigation in population-level health trajectories.

### 2.6. Dietary Interventions Can Lower the Percentage of n-6 in HUFA, with Potential Health Benefits and Associated Reductions in Healthcare Costs

Dietary modification of the relative intake of *n*-6 and *n*-3 PUFAs has been shown to reliably shift the HUFA composition of membrane phospholipids. Because the incorporation of HUFAs follows competitive, substrate-driven kinetics, reducing dietary linoleic acid or increasing the intake of long-chain *n*-3 fatty acids leads to a measurable decline in the proportion of *n*-6 HUFAs in blood and tissue lipids. Lands demonstrated that such shifts occur in a predictable, dose-dependent manner and can be monitored quantitatively through the *n*-6 HUFA Score, which reflects the integrated biochemical impact of dietary patterns on membrane composition [3].

These diet-induced changes in HUFA composition are associated with physiologically meaningful benefits. A lower percentage of *n*-6 in HUFA reduces the relative availability of arachidonic acid for the formation of more potent pro-inflammatory lipid mediators, while increasing the contribution of EPA- and DHA-derived metabolites that are generally less inflammatory in character. Calder and colleagues have shown that such shifts in HUFA balance can modulate inflammatory tone, improve endothelial and immune function, and support healthier metabolic regulation [27,28]. Clinical and nutritional studies further indicate that increasing long-chain *n*-3 intake contributes to improved health trajectories in aging and chronic disease contexts, underscoring the relevance of HUFA modulation as a practical intervention strategy [29].

From a public-health perspective, these biochemical and physiological improvements may have potential economic implications. Because chronic inflammatory and metabolic diseases represent major drivers of healthcare expenditure, even modest population-level improvements in the membranal HUFA composition could reduce the incidence or severity of these conditions. Lands therefore argues that dietary interventions targeting the *n*-6/*n*-3 HUFA balance represent a low-cost, scalable, and mechanistically grounded opportunity to reduce long-term healthcare burdens [3]. While the mechanistic logic is coherent, further corroboration is required—not least because the potential health and economic implications make such additional evaluation imperative (see also Section 2.13).

### 2.7. The Individual n-6 HUFA Profile Serves as a Valuable Surrogate Biomarker Because It Reflects Both Dietary Inputs and Pathophysiological Outcomes

The proportion of *n*-6 HUFAs within the total HUFA pool—expressed as the percentage of *n*-6 in HUFA measured in blood or tissue lipids—provides a quantitative readout of an individual’s HUFA balance. This metric reflects the competitive incorporation of dietary *n*-6 and *n*-3 precursors into membrane phospholipids and thereby influences the intensity of *n*-6-derived eicosanoid formation. Lands emphasized that the percentage of *n*-6 in HUFA is tightly linked to dietary PUFA patterns and correlates with a wide range of physiological and pathophysiological outcomes [2,4,30].

Individuals whose HUFA profile contains less than 50% *n*-6 HUFAs are more likely to release *n*-3 HUFAs such as EPA upon activation of cytosolic phospholipase A_2_, resulting in a greater proportion of *n*-3-derived lipid mediators. This shift reflects the substrate-driven nature of HUFA metabolism and the competitive dynamics that shape access to the major eicosanoid-producing pathways. By contrast, the average HUFA profile in the United States is approximately 75–80% *n*-6 in HUFA [31], indicating a biochemical environment dominated by arachidonic-acid-derived metabolites. Such a profile increases the likelihood of producing more potent *n*-6-derived mediators and has been associated with adverse interactions affecting metabolic, immunologic, and organ-specific functions [5].

Because it integrates dietary inputs, enzymatic competition, and downstream mediator production, the percentage of *n*-6 in HUFA serves as a robust surrogate biomarker of both nutritional status and inflammatory propensity. It captures the mechanistic link between diet, membrane composition, and physiological outcomes, making it a valuable tool for assessing health trajectories within Lands’ framework.

### 2.8. Combining Reduced n-6 with Increased n-3 PUFA Intake Most Effectively Lowers the Percentage of n-6 in HUFA, Owing to the Predictable Quantitative Dynamics of the Competing HUFA Families

The available evidence indicates that dietary strategies combining a reduction in *n*-6 PUFA intake with an increase in *n*-3 PUFA intake are the most effective means of lowering the percentage of *n*-6 in HUFA. Lands emphasized that the HUFA profile responds in a predictable, competitive manner to changes in dietary precursor supply, and that combined interventions shift the HUFA balance more efficiently than increasing *n*-3 intake alone [2]. This principle is consistent with classical dose–response studies showing that the relative abundance of competing essential fatty acids determines their proportional incorporation into tissue lipids [32].

Reducing dietary *n*-6 PUFAs decreases the competitive pressure exerted by linoleic-acid-derived metabolites, thereby allowing *n*-3 PUFAs to displace *n*-6 HUFAs more effectively within membrane phospholipids. As a result, the percentage of *n*-6 in HUFA declines more rapidly, lowering the substrate availability for excessive *n*-6-derived eicosanoid formation. This shift in HUFA composition reduces the likelihood of overactive arachidonic-acid-mediated responses and supports a more balanced mediator profile.

These predictable quantitative dynamics of competing *n*-3 and *n*-6 PUFA families provide a mechanistic foundation for designing preventive nutritional strategies. By simultaneously lowering dietary *n*-6 intake and increasing *n*-3 intake, it becomes possible to achieve more favorable HUFA profiles with greater efficiency and potentially greater health benefits than through *n*-3 supplementation alone.

### 2.9. Failure to Account for the Population-Wide Oversupply of n-6 PUFAs May Help Explain Inconsistent Results in Randomized Controlled Trials Evaluating the Clinical Efficacy of n-3 PUFAs

The inconsistencies observed between epidemiological findings and randomized controlled trials (RCTs) on the benefits of *n*-3 PUFAs may stem from the fact that many RCTs increased *n*-3 intake without accounting for the pre-existing oversupply of *n*-6 PUFAs characteristic of Western dietary patterns. If this interpretation is correct, a substantial number of existing RCTs on *n*-3 PUFA supplementation may require reevaluation, as their design did not consider the competitive metabolic dynamics that determine HUFA composition.

Observational evidence supports this concern. Populations in which less than 50% of total HUFAs are *n*-6 HUFAs tend to exhibit lower prevalence of chronic diseases compared with populations whose HUFA profiles exceed 50% *n*-6 HUFAs [33]. This threshold reflects the point at which cytosolic phospholipase A_2_ is more likely to encounter *n*-3 HUFAs than arachidonic-acid-derived *n*-6 HUFAs, thereby shifting mediator production toward less inflammatory profiles. Based on such findings, Lands proposed that consuming foods that help maintain less than 50% *n*-6 in HUFA constitutes an effective strategy for primary disease prevention—and potentially for therapeutic benefit as well [4].

### 2.10. Measures of Basal as Well as Final n-6 and n-3 HUFA Status Should Be Considered Important and Valid Biomarkers for Designing and Monitoring Effective Nutritional Strategies

Assessing *n*-6 or *n*-3 HUFAs in isolation provides only a partial and potentially misleading picture of the physiological consequences of dietary choices. Because *n*-6 and *n*-3 HUFAs compete directly for incorporation into membrane phospholipids and for access to the major eicosanoid-producing pathways, their relative abundance—rather than their absolute levels—determines the pattern and intensity of downstream lipid-mediator production. The HUFA balance, expressed as the percentage of *n*-6 in total HUFAs, therefore offers a more integrative and mechanistically meaningful indicator of an individual’s biochemical state.

Within this framework, it is essential to distinguish between two related but conceptually different metrics: the % *n*-6 in HUFA and the *n*-6 HUFA Score. The % *n*-6 in HUFA is a calculated proportion that reflects the competitive distribution of *n*-6 and *n*-3 HUFAs within membrane phospholipids and predicts the likely substrate availability for eicosanoid formation. In contrast, the *n*-6 HUFA Score is a measured biomarker derived from blood or tissue lipid analyses. It represents the actual, integrated HUFA composition of an individual at a given time and therefore captures both long-term dietary patterns and ongoing metabolic processes. While the % *n*-6 in HUFA provides the mechanistic logic of HUFA competition, the *n*-6 HUFA Score quantifies its realized biochemical outcome in vivo.

Because these two measures are complementary, both should be assessed at the beginning and end of any nutritional intervention aimed at modifying HUFA balance. The % *n*-6 in HUFA allows researchers to predict the expected direction and magnitude of change based on dietary design, whereas the *n*-6 HUFA Score provides the empirical verification of whether the intended biochemical shift has actually occurred. Lands and colleagues emphasized that this dual approach captures the dynamic interactions of dietary intake, enzymatic competition, and mediator biosynthesis, making these metrics robust surrogate biomarkers for predicting physiological outcomes and monitoring the effectiveness of nutritional strategies [10].

Together, the % *n*-6 in HUFA and the *n*-6 HUFA Score provide a coherent and mechanistically grounded framework for designing, implementing, and evaluating preventive and therapeutic nutrition interventions.

### 2.11. A Range of Non-Communicable Diseases Appears to Be Associated with Elevated n-6 HUFA Levels, and the Underlying Pathophysiological Mechanisms Are Increasingly Understood

A growing body of evidence indicates that elevated levels of *n*-6 HUFAs in membrane phospholipids are associated with the development and progression of numerous non-communicable diseases. Lands emphasized that excessive *n*-6 HUFA availability may amplify immune-suppressive and pro-inflammatory signaling, particularly through arachidonic-acid-derived eicosanoids [3]. These mediators promote the recruitment of immune cells such as monocytes to sites of tissue stress or injury and activate them to release additional cytokines, chemokines, and lipid mediators. This might create a self-reinforcing cycle in which inflammatory cell accumulation and mediator production could be seen as perpetuating and potentially intensifying local inflammation.

Over time, such a positive feedback loop might be capable of transforming a small, localized inflammatory focus in adipose tissue, the vasculature, the liver, the lung, or the myocardium into a site that could come to display abnormal cellular composition and impaired function. Such destabilizing molecular and cellular processes are characteristic of many chronic and acute health conditions, including metabolic, cardiovascular, pulmonary, and immune-related disorders [3].

In contrast, *n*-3 HUFAs modulate these processes through several complementary mechanisms. By reducing the relative abundance of arachidonic acid in membranes, EPA and DHA limit substrate availability for the formation of more potent *n*-6-derived eicosanoids. In addition, both fatty acids give rise to less inflammatory metabolites and to specialized pro-resolving mediators (SPMs), which actively promote the resolution of inflammation and the restoration of tissue homeostasis [7,34,35,36]. These mechanistic insights underscore the central importance of HUFA balance in preventing and controlling conditions in which dysregulated or excessive inflammation is a contributing risk factor. However, while these mechanisms provide biological plausibility, they do not in themselves establish that elevated *n*-6 HUFA levels causally drive the onset of specific non-communicable diseases. However, against the background alluded to, further empirical investigation would appear warranted, particularly given the potential epidemiological significance should future studies yield consistent and corroborating findings.

### 2.12. In Cardiovascular Disease (CVD), Preliminary Evidence Already Suggests a Potential Causal Role for an Increased n-6 HUFA Profile

Cardiovascular disease is a progressive chronic inflammatory condition that often begins with transient oxidative insults and endothelial dysfunction induced by excessive postprandial energy intake [3]. These insults occur preferentially at vascular sites exposed to disturbed flow, such as bifurcations, where low and oscillatory shear stress promotes the local accumulation of inflammatory cells and mediators [37,38,39,40]. Repeated exposure to such transient dysfunction, together with decades of continued recruitment of inflammatory cells, might be seen as potentially contributing to the gradual development of clinically manifest CVD in the population [2].

Lands proposed that much of this transient endothelial dysfunction may be reversible unless it is mechanistically amplified by *n*-6 HUFA-facilitated eicosanoid signaling, which enhances the recruitment and activation of inflammatory cells [2]. Several *n*-6 HUFA-derived lipid mediators exhibit more potent inflammatory actions than their *n*-3 HUFA-derived analogs [41]. A well-characterized example is leukotriene B4 (LTB4), which strongly promotes monocyte adhesion to the vascular endothelium [42]. This mechanism could help explain why the resolution of postprandial endothelial dysfunction appears impaired in individuals whose HUFA profile contains approximately 80% *n*-6 HUFAs, whereas such impairment appears reduced in individuals with less than 50% *n*-6 in HUFA [33].

These observations support the hypothesis that an elevated percentage of *n*-6 in HUFA may contribute to inflammatory processes relevant to CVD pathogenesis [10]. However, this remains a mechanistic and observational hypothesis rather than a demonstrated causal relationship. Lands argued that blood cholesterol levels predict cardiovascular mortality only to the extent that *n*-6 HUFAs quantitatively exceed *n*-3 HUFAs, highlighting the importance of HUFA balance in modulating risk [4,30]. From this perspective, the widespread consumption of diets providing more than 2% of energy as linoleic acid (LA) could theoretically contribute to harmful inflammatory actions that promote various non-communicable diseases, including CVD [43].

However, epidemiological evidence remains mixed. A large U.S. cohort study reported that higher circulating LA levels were associated with lower all-cause and coronary heart disease mortality [44], and a pooled analysis of 30 prospective cohort studies found that higher circulating LA was associated with lower risks of total CVD, cardiovascular mortality, and ischemic stroke [45]. These findings contrast with mechanistic concerns about excessive *n*-6 HUFA availability and illustrate that this remains an area of active scientific debate [46,47,48]. Thus, while mechanistic models suggest potential pathways through which *n*-6 HUFAs could influence CVD risk, population-level evidence does not yet provide a consistent causal picture.

Taken together, these observations suggest that conventional treatment strategies—such as statin therapy, which effectively reduces plaque burden but does not modify HUFA balance—may not fully address the underlying biochemical drivers of CVD risk. If future research confirms that an elevated *n*-6 HUFA profile contributes causally to impaired endothelial recovery and chronic vascular inflammation, then nutritional strategies targeting HUFA balance may represent an important, yet underappreciated, component of comprehensive cardiovascular prevention.

### 2.13. Achieving An n-6 HUFA Percentage near 50% May Help Reduce Annual Healthcare Expenditures and Improve the Cost-Effectiveness of Public Health Interventions

The mechanistic principles outlined in Section 2.1, Section 2.2, Section 2.3 and Section 2.4 establish how competitive HUFA dynamics shape the biochemical environment in which inflammatory and regulatory processes unfold. These mechanisms provide the foundation for understanding how shifts in dietary PUFA patterns can influence physiological function at the population level. Section 2.13 therefore extends this mechanistic framework to its broader public-health implications, examining how HUFA balance may intersect with chronic disease burden, healthcare utilization, and the potential economic impact of preventive strategies.

In the United States, the ten chronic conditions associated with the greatest health-related financial losses in occupational medicine include depression, obesity, arthritis, back and neck pain, anxiety, gastroesophageal reflux disease, allergy, cancer, chronic pain, and hypertension [49]. Many of these conditions have been linked to unwanted HUFA-mediated or *n*-6 HUFA-derived eicosanoid actions, which can amplify inflammatory signaling and contribute to disease progression. Despite this, current healthcare programs rarely consider the possibility that excessive *n*-6 HUFA-derived mediator activity may worsen chronic health conditions, or that lowering the *n*-6 HUFA balance could reduce the intensity of these unwanted actions [50]. These links, however, are largely associative and mechanistic; causal relationships remain to be established.

Nevertheless, building on these mechanistic and observational considerations, evidence from workplace health analyses suggests that employees with lower percentages of *n*-6 in HUFA may exhibit reduced annual healthcare claim costs and improved cost-effectiveness of health interventions [50]. While these findings are observational and cannot establish causality, they suggest that achieving an HUFA profile closer to 50% *n*-6 may not only support better physiological outcomes but also reduce economic burdens associated with chronic disease management.

To facilitate such improvements, prevention programs could incorporate freely available tools such as *n*-3/*n*-6 balance scores and menu-planning applications designed to help individuals adjust their dietary choices toward a more favorable HUFA balance [51]. By enabling populations to shift food intake patterns in a direction that lowers the risk of *n*-6 HUFA-mediated disorders, these tools may contribute to improved health status and quality of life. Moreover, such strategies may offer a substantial return on investment, particularly when integrated into broader public health or workplace wellness initiatives [52]. Nonetheless, the economic implications of modifying HUFA balance require further empirical evaluation before firm conclusions can be drawn.

## 3. What Evidence-Based Concepts Support Lands’ Hypotheses?

As summarized in Table 2, several evidence-based concepts support Lands’ hypotheses and clarify why HUFA balance is central to understanding diet-related health outcomes. These principles show that PUFAs act as biologically active signaling molecules, that dietary patterns strongly determine the body’s *n*-6/*n*-3 HUFA profile, and that the marked rise in linoleic acid intake in recent decades has shifted this balance toward *n*-6 dominance. As a result, the availability of *n*-3 HUFAs has declined in individuals consuming Western dietary patterns, with broad implications for the development of non-communicable diseases. Together, these insights highlight that optimal *n*-3 HUFA requirements depend on an individual’s existing *n*-6 HUFA burden and establish HUFA balance as a key target for preventive nutritional strategies.

### 3.1. The n-6/n-3 HUFA Balance Governs Inflammatory, Immunologic, and Metabolic Signaling

**Figure 6:**
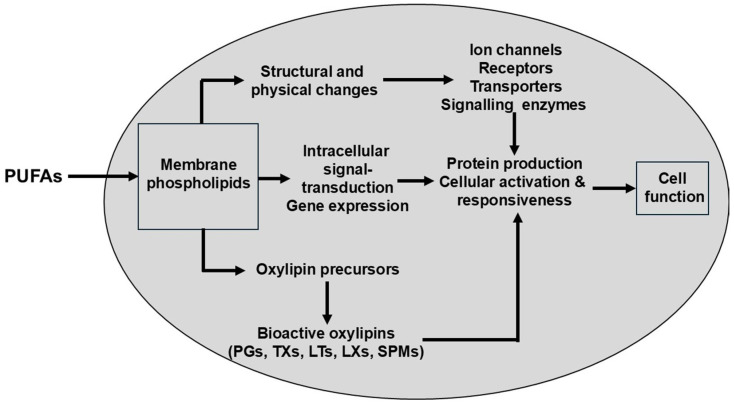
Overview of the mechanisms by which polyunsaturated fatty acids (PUFAs), especially highly unsaturated fatty acids, regulate cell function. LT, leukotriene; LX, lipoxin; PG, prostaglandin; SPM, specialized pro-resolving mediator; TX, thromboxane.

PUFAs and HUFAs are more than metabolic fuel; they can be considered as pharmacologically active substances provided by nutrition [53,54,55,56,57]. Possible mechanisms by which PUFAs can act as regulators of cell function are shown in Figure 6.

HUFAs are precursors to potent lipid mediator signaling molecules (oxylipins), also termed “eicosanoids” or “docosanoids”, which have important roles in regulating inflammation, immune function, thrombosis, and smooth muscle contraction, amongst others. This was recently reviewed with regard to the role of eicosanoids in liver repair, regeneration, and cancer [58]. Likewise, relatively recently, the so-called “specialized pro-resolving mediators” or “SPMs” (protectins, resolvins, maresins, and lipoxins) were discovered. Their nomenclature was coined because of their intimate involvement in the resolution of inflammation [34]. Their therapeutic potential in preventing and treating inflammatory disorders has already been reviewed repeatedly [36,59,60,61]. Nevertheless, some question the relevance of these mediators [62,63]. However, it is likely that in vivo oxidation of HUFAs by enzymatic or non-enzymatic processes produces potent signaling molecules [64].

Beyond acting as substrates for the synthesis of bioactive lipid mediators, PUFA (and HUFA) availability plays an important role in the composition of all cell membranes, influencing membrane fluidity and thus membrane protein- and receptor function [65], although recent analyses caution that fluidity alone may not account for the full spectrum of PUFA-dependent membrane effects.

Most importantly, however, PUFAs also affect intracellular signal-transduction and gene expression, e.g., via influencing the activity of transcription factors including NFk-B (nuclear factor kappa-light-chain-enhancer of activated B-cells), PPARs (peroxisome proliferator-activated receptors) and SREBPs (sterol regulatory element-binding proteins) [66]. The regulation of gene expression by fatty acids is believed to be one of the most important factors impacting the development of many non-communicable diseases.

The transcription factor NFk-B is crucial in controlling inflammatory signaling pathways as it regulates several cytokines, chemokines, adhesion molecules and inducible effector enzymes like cyclooxygenase-2 [67]. NFk-B is regulated by PUFAs (amongst many other stimuli), with EPA and DHA being demonstrated to decrease NFk-B activation [68,69,70]. Many of the *n*-3 PUFA-related anti-inflammatory actions are based on the inhibition of the NFk-B activity as described elsewhere [71]. However, this effect is not observed to the same extent with *n*-6 PUFAs. Thus, an oversupply of *n*-6 PUFAs (in relation to *n*-3 PUFAs) allows an increased activity of NFk-B [72], and the creation of a pro-inflammatory state.

PPARs (α, β/δ, and γ) are ligand-activated nuclear transcription factors [73]. Endogenous ligands for PPARs include PUFAs (among others), especially those of the *n*-3 family and their eicosanoid derivatives [74]. The results of a meta-analysis recently indicated a significant elevation in PPAR-γ and PPAR-α gene expression due to *n*-3 PUFA supplementation [75]. PPARs have been shown to influence inflammation, cardiac and retinal disorders, pregnancy, the homeostasis of lipids and cancer. Although the different PPARs have different tissue distributions, their biological functions overlap [76]. PPAR-γ exhibits anti-diabetic and anti-atherosclerotic effects in adipocytes and skeletal muscles; PPAR-α controls metabolism, exerting multiple effects in the liver, heart, and vessel wall; and PPAR-β is expressed ubiquitously in the body and controls the expression of genes involved in glucose and lipid metabolism. PPAR biology is reviewed in full elsewhere [74]. PPARs have been shown to inhibit NFk-B activation and, therefore, play an important role in regulating several inflammatory processes [77]. Indeed, both EPA and DHA downregulate lipopolysaccharide-induced activation of NFk-B partly via a PPAR-dependent pathway [78]. Moreover, PPAR-α has been shown to exert hypolipidemic effects through activation of genes encoding proteins involved in lipid oxidation [79]. Thus, PPARs, particularly PPAR-α, play an important role in insulin sensitization, atherosclerosis protection, and metabolic disease resolution [80]. *n*-3 PUFAs stimulate the oxidative metabolism of fatty acids by the PPAR-α mediated pathway [81] and this partly explains the observations that *n*-3 PUFAs lower blood triglyceride concentrations by 20–30% [82,83]. This is accompanied by a moderate rise in HDL-cholesterol, mainly by influencing HDL remodeling and by promoting hepatobiliary sterol excretion [84]. As reviewed elsewhere [79], the *n*-3 fatty acids EPA and DHA are more potent as in vivo activators of PPAR-α than the *n*-6 PUFAs. Thus, an oversupply of *n*-6 PUFAs relative to *n*-3 PUFAs not only permits increased activity of NFk-B but also does not favour activation of PPAR-α.

SREBP-1 is a transcription factor that regulates expression of genes involved in lipid and fatty acid metabolism, such as fatty acid synthase (FAS). SREBP-1 is required for the insulin-mediated induction of hepatic fatty acid and triglyceride synthesis. PUFAs have been shown to suppress SREBP-1c gene expression and so inhibit transcription of hepatic genes involved in lipid biosynthesis [83,85]. This reduces lipid accumulation within the liver [86,87,88]. Of note, however, *n*-3 PUFAs are more potent inhibitors of SREBP-1c than *n*-6 PUFAs [89]. Thus, an imbalanced oversupply of *n*-6 PUFAs does not permit sufficient inhibition of SREBP-1c, leading to hepatic lipid accumulation.

**Figure 7:**
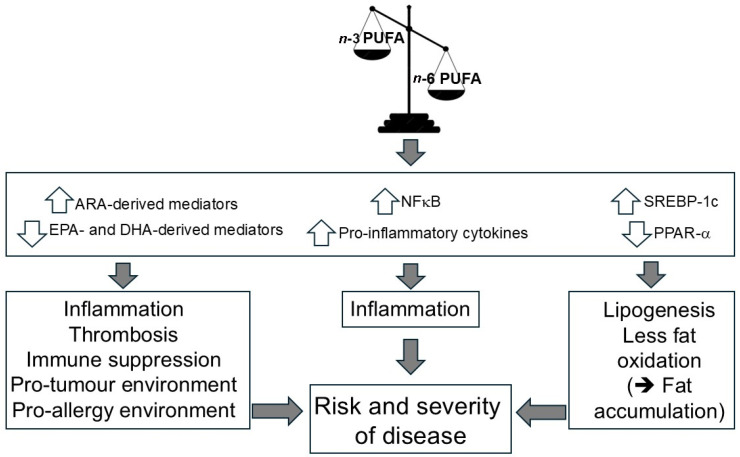
Overview of the mechanisms by which an unbalanced *n*-6 and *n*-3 PUFA dietary intake and status affect disease risk and severity. NFk-B, nuclear factor kappa-light-chain-enhancer of activated B-cells; PPAR, peroxisome proliferator-activated receptor; SREBP, sterol regulatory element-binding protein; up arrow = quantitative availability or activity increased; down arrow = quantitative availability or activity decreased.

In Figure 7, the consequences of an unbalanced dietary intake of *n*-6 and *n*-3 PUFAs are depicted.

As reviewed by Patterson et al. [79], excessive intake of *n*-6 PUFAs (e.g., linoleic acid) reduces the conversion of ALA into its *n*-3 HUFA derivatives, including EPA and DHA. The resulting increase in membrane phospholipid arachidonic acid (ARA) content promotes greater production of ARA-derived eicosanoids and fewer EPA- and DHA-derived eicosanoids and docosanoids. Consequently, pro-inflammatory and immunosuppressive mediators predominate over their anti-inflammatory and immune-augmenting counterparts, creating a biochemical environment that favors inflammation accompanied by impaired immune function.

In addition, the relative deficiency of *n*-3 HUFAs leads to reduced PPAR-α expression and activation. At the same time, the predominance of *n*-6 PUFAs limits the suppression of SREBP-1c and NFκB activity. Together, these effects increase lipogenesis and decrease fatty-acid oxidation, thereby promoting hepatic steatosis alongside inflammation and immune suppression.

As a result, dysfunction emerges at the cellular, organ, and systemic levels, contributing to clinical manifestations across disorders of lipid metabolism, inflammation, and infection.

**Figure 8:**
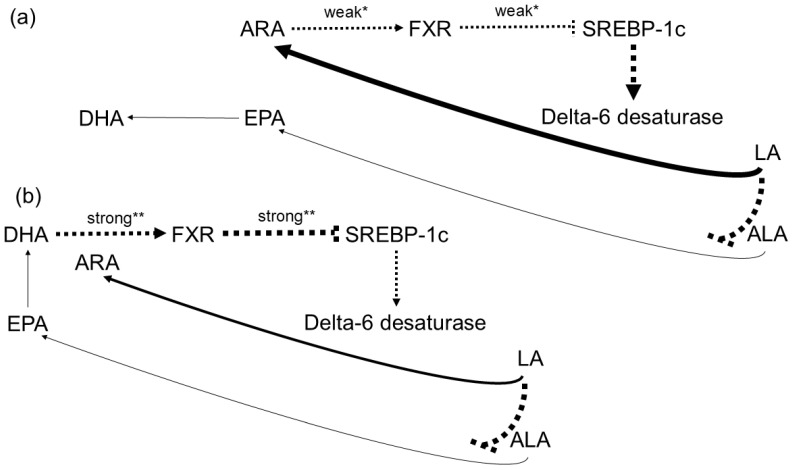
Depiction of the actions of ARA (**a**) and DHA (**b**) on delta-6 desaturase activity. (**a**) ARA is a weaker (*) agonist of FXR than DHA, meaning weaker (*) inhibition of SREPB-1c. SREBP-1c promotes delta-6 desaturase, allowing LA conversion to ARA. LA competitively inhibits ALA metabolism. (**b**) DHA is a stronger (**) agonist of FXR than ARA, meaning stronger (**) inhibition of SREPB-1c. SREBP-1c less strongly promotes delta-6 desaturase so decreasing LA conversion to ARA but increasing ALA conversion to EPA since LA competitively inhibits ALA metabolism less strongly. Dashed lines indicate regulation; full lines indicate metabolic conversion; solidity of full lines indicates metabolic flow rate. FXR, farnesoid X receptor; SREBP, sterol receptor element-bonding protein.

Interestingly, the impact of dietary PUFAs on gene expression (via transcription factors) is also believed to regulate the activity of Δ6-desaturase (Figure 8), the rate-limiting enzyme in the PUFA biosynthetic pathway (Figure 1).

A key driver of transcription of the Δ6-desaturase gene (i.e., *FADS2*) appears to be SREBP-1c [90]. Conversely, the farnesoid X receptor (FXR) suppresses the expression of mature SREBP-1c at both the transcriptional and post-translational levels (reviewed in [91]). Although generally regarded as a bile acid receptor, FXR can also be activated by certain PUFAs. The affinities of ARA, DHA, and ALA for FXR have been reported as 2.6, 1.5, and 3.5 µM, respectively [92].

The comparatively weaker FXR activation by ARA—relative to DHA—results in less suppression of SREBP-1c, thereby permitting higher Δ6-desaturase activity and consequently greater metabolic throughput from LA to ARA (Figure 8a). This mechanism can be understood as a positively reinforcing feedback loop driven by increased *n*-6 PUFA availability.

By contrast, this framework may also help explain, at least in part, the down-regulatory effects of *n*-3 PUFAs (compared with *n*-6 PUFAs) on serum triglycerides: *n*-3 PUFA-mediated activation of FXR suppresses SREBP-1c transcription more effectively, the latter being a key driver of lipogenesis (see above). Likewise, diets rich in ALA and DHA would be expected to decrease Δ6-desaturase expression by down-regulating SREBP-1c activity (Figure 8b) [91].

### 3.2. Excessive n-6 PUFA and HUFA Abundance Drives Molecular, Cellular, and Organ-Level Pathomechanisms Linked to Chronic Disease

**Figure 9:**
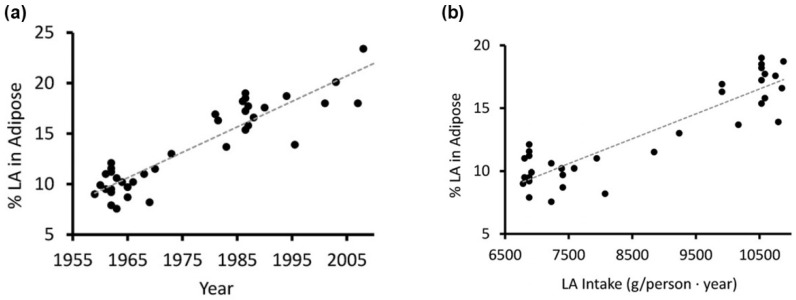
Increase in adipose tissue linoleic acid (LA) concentration (% of total fatty acids) over time by the year 1959 to 2008 (R2 = 0.83; *p* 0.001) (**a**) and (**b**) correlation between adipose tissue LA concentration and annual dietary LA intake (g/person) by the year 1959 to 1999 (R2 = 0.81; *p* 0.001), among US adults. Adipose tissue LA concentration is strongly correlated with dietary LA intake. The figures are taken from Guyenet and Carlson [95] (Open Access CC BY-NC-ND 4.0).

It is well known that cell and tissue phospholipid *n*-6 and *n*-3 fatty acids are modified by dietary *n*-6 and *n*-3 fatty acid intakes (see Section 3.1). With this in mind, it is remarkable that the consumption of LA-rich vegetable oils, especially soybean oil, skyrocketed in the 20th century. Assumably, increased consumption of LA-rich vegetable oils has led to a marked increase in LA intake such that LA now represents about 90% of PUFAs in many Western diets compared with 50% before the early 1990s [93,94]. Accordingly, an increase in adipose tissue LA concentration over time (for the years 1959–2008) across all subcutaneous sites measured was shown (Figure 9a) [95].

Moreover, it could be demonstrated that adipose tissue LA concentration is strongly correlated with dietary LA intake (expressed as g/person x year) for the years 1959–1999. (Figure 9b). In circulating cells of individuals consuming a Western diet, *n*-6 PUFAs and ARA are greatly in excess of EPA and DHA; for example, LA and ARA may comprise approximately 10% and 20% of total fatty acids, respectively, whereas EPA and DHA contribute about 0.5% and 2.5%. This fatty-acid pattern corresponds to a % *n*-6 in HUFA of roughly 85%.

While the historical intake data presented here illustrate the long-term trajectory of dietary shifts, more recent datasets (e.g., NHANES and FAOStat) similarly indicate persistently high linoleic-acid availability, even though they do not provide biomarker-based information relevant to the mechanistic focus of this review.

Given that PUFAs, HUFAs and their oxylipin derivatives regulate multiple molecular, cellular and organ systems [6,67,71,83,96,97,98,99,100,101,102,103,104,105,106,107,108,109,110,111,112,113,114,115,116,117,118,119,120,121,122,123,124,125,126,127,128,129,130,131,132,133,134,135,136,137] (Table 3) and that these are mostly optimized by *n*-3 HUFAs, it is likely that an excessively high abundance of *n*-6 PUFAs and HUFAs as a result of a dietary imbalance can promote several different morbidities (Table 4). As indicated previously, this situation can be improved by lowering the intake of *n*-6 PUFAs and increasing the intake of *n*-3 PUFAs.

While there may be a link between an endogenous excess of *n*-6 PUFAs and the presence of many non-communicable diseases, we still know relatively little detail about the pathomechanisms involved. As already mentioned, pro-inflammatory and immunosuppressive effects, as well as “lipogenic actions” associated with the deposition of “ectopic fat” and the development of “lipotoxicity,” seem to play an important role. Oxidative processes that are related to an overabundance of LA and are probably also of pathogenetic significance, as recently reviewed [46]. In fact, there is a U-shaped curve when considering the effects of increasing availability of LA. A modest, evolutionarily consistent intake of LA has been associated with a decreased risk of various non-communicable diseases, whereas highly elevated levels of LA that result from high dietary intake are proposed to be associated with an increased disease incidence. An important causal factor seems to be that LA becomes a precursor to oxidized LA metabolites (OXLAMs), such as 4-hydroxynonenal (HNE), 9- and 13-hydroxy octadecadienoic acid (9- and 13-HODE), and 9- and 13-oxo-octadecadienoic acid (9- and 13-oxoODE) [138]. Moreover, and again as reviewed elsewhere [46], LA conversion may also lead to the formation of free radicals, such as 8-hydroxyoctanoic acid and heptanoic acid [139]. In addition, LA may be further metabolized into ARA, which is a precursor to oxidized ARA metabolites (OXAAMs), including 5-, 8-, 9-, 11-, 12-, and 15-hydroxy-eicosatetraenoic acid (HETE) [140]. The increased circulation of LA or ARA-related oxidized metabolites and free radicals has been linked to different types of diseases (e.g., cardiovascular, atherosclerotic, hepatic, etc.) [141].

Evolutionary analyses suggest that humans historically consumed LA and ALA in a ratio close to 1:1 [142], whereas current German nutritional guidelines imply a ratio of approximately 5:1 [143]. However, dietary changes over the past few decades have led to striking increases in the intake of *n*-6 PUFAs, especially LA, resulting in dietary LA-to-ALA ratios exceeding 10:1 and reaching up to 20:1 in some Western diets [144], despite broad recognition that such an imbalance may contribute to the development of various chronic diseases [145].

### 3.3. Increasing Linoleic Acid Intake May Amplify HUFA-Mediated Pathomechanisms in n-6–Dominant Physiological States

The mechanistic considerations in Section 3.1 and Section 3.2 underscore that *n*-6-dominant HUFA pools provide a biochemical environment in which several pathomechanisms are more readily amplified. This perspective makes the historical rise in dietary LA intake particularly consequential.

**Figure 10:**
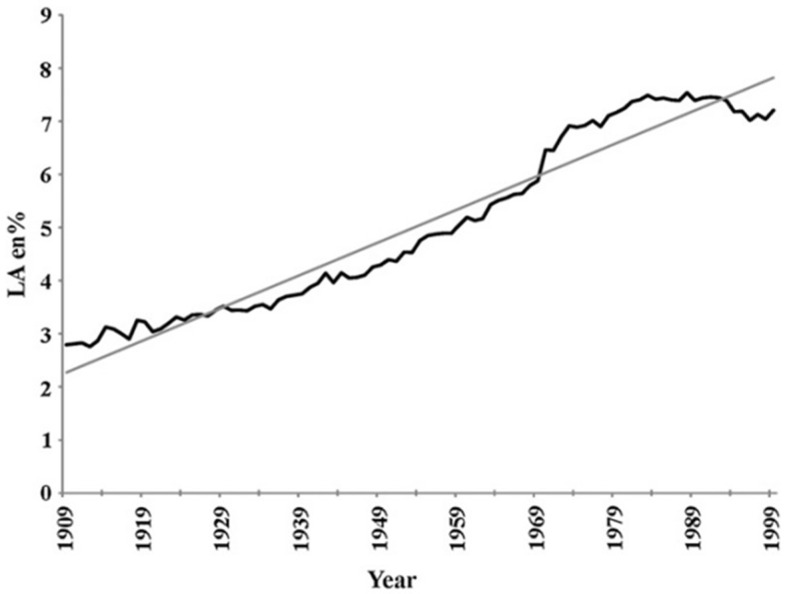
Estimated availability of linoleic acid (LA) as a percentage of dietary energy in the adult US diet from 1909 to 1999. Reprinted from [94]: The American Journal of Clinical Nutrition, Vol 93, T.L. Blasbalg et al., Changes in consumption of omega-3 and omega-6 fatty acids in the United States during the 20th century, Pages 950–962, Copyright (2011), with permission from The American Society for Nutrition.

As already described, the LA content of adipose tissue in US adults has increased markedly since the late 1950s [46,95], reflecting a substantial rise in dietary LA intake over recent decades [94]. Blasbalg et al. [94] provided a detailed historical analysis of PUFA consumption in the United States, showing that soybean oil intake increased more than 1000-fold from 1909 to 1999. Over the same period, LA availability rose from 2.79% to 7.21% of total energy intake (Figure 10), and the dietary LA-to-ALA ratio increased from 6.4 to 10.0 [94]. These shifts were largely driven by changes in food production and the widespread adoption of industrial vegetable oils in processed foods.

Together, these developments have progressively increased the proportion of *n*-6-derived HUFAs in tissues, thereby reinforcing the *n*-6-dominant physiological states in which the HUFA-mediated pathomechanisms outlined in Section 3.1 and Section 3.2 are most likely to be amplified.

The historical estimates of linoleic-acid availability presented here illustrate the long-term dietary shift relevant to Lands’ framework; although these data do not include HUFA measurements, more recent datasets likewise report only intake or availability and therefore complement—but do not supersede—the evidence used in this section.

### 3.4. The Concept of a Dietary Toxicity Threshold for Linoleic Acid Appears to Be Supported by Available Evidence, Yet Remains Debated

Questions about the optimal dietary supply of LA remain unresolved. LA is an essential fatty acid, and deficiency symptoms occur at low intakes. According to official reports, the minimum intake of essential fatty acids is 2.5%E LA plus 0.5%E ALA to avoid deficiency symptoms in adults [146]. Other experts, however, have argued that it may be reasonable to keep dietary LA availability closer to the minimum amount required to prevent essential fatty acid deficiency symptoms [147]. This level is generally assumed to be 1–2% of energy intake [148], and Holman’s work in infants indicated that 1.4%E LA was sufficient [149]. At the other end of the spectrum, some experts have estimated that a practical toxicity threshold for dietary LA is around 4%E [94]. According to Lands [2], explicit data for LA are consistent with a Recommended Dietary Allowance (RDA) near 0.5%E—meeting the needs of 97–98% of individuals—and a Tolerable Upper Intake Level (UL) near 2%E, with no likely risk of adverse health effects.

These more cautious views have, however, largely not been incorporated into major guideline documents. In 2009, the American Heart Association (AHA) recommended consuming at least 5–10% of energy from *n*-6 PUFAs (mainly as LA), suggesting that this strategy would reduce the risk of CHD relative to lower intakes [150]. The committee further stated that even higher intakes appeared safe and might be even more beneficial as part of a low-saturated-fat, low-cholesterol diet. Accordingly, it concluded that reducing *n*-6 PUFA intakes from their then-current levels would be more likely to increase than to decrease the risk of CHD. A year later, the AHA science advisory was reviewed and reaffirmed [151]. This position was subsequently questioned and challenged [152] in light of data from Ramsden and colleagues [43,153]. Since that scientific exchange, explicit controversy over upper limits for *n*-6 PUFAs has become less visible, with only a few important contributions by Lands—some of which have already been discussed—continuing to highlight the possibility of *n*-6 PUFA overload [2,33]. More recently, concerns about excessive *n*-6 PUFA exposure have reemerged [46,145].

Nevertheless, follow-up guidelines from the AHA have not explicitly abandoned the recommendation of relatively high *n*-6 PUFA intakes [154]. Based on their recommendations to reduce saturated fat intake and optimize overall caloric intake, a higher intake of monounsaturated and polyunsaturated fats is still advocated, without explicitly addressing the importance of a balanced ratio of LA and ALA. In Europe, the European Food Safety Authority suggests a reference intake for LA of 10 g/day (approximated as 4%E) [155], which is somewhat lower than the recommendations made in the United States. The United Kingdom recommends that *n*-6 PUFAs (mainly LA) comprise up to 6%E [156]. It is also worth noting that in 1994, the UK regulatory authority commented that “the safety of average intakes by the population of *n*-6 PUFAs over about 6% of energy [156] remains untested,” that “diets providing more than about 6% of energy as *n*-6 PUFAs cannot currently be recommended,” and that “there is reason to be cautious about high intakes of *n*-6 PUFAs and we recommend that the proportion of the population consuming more than about 10% of energy [as *n*-6 PUFAs] should not increase” [157]. The recent 2023 AHA guideline, which continues to endorse higher intakes of polyunsaturated fats as a replacement for saturated fats, thus stands in contrast to these earlier cautionary statements. The most recent AHA dietary guidance (2025) continues to emphasize overall dietary patterns and food quality but does not introduce new recommendations regarding PUFA, *n*-6 PUFA, or LA intake, nor does it address potential upper limits or the relevance of LA–ALA balance.

Taken together, current recommendations for dietary LA intake vary strikingly across institutions and experts, as shown in Table 5. This wide divergence underscores the ongoing uncertainty about the optimal range of LA intake and highlights the need for clearer guidance that integrates both essentiality and HUFA-mediated physiological considerations.

Basic research—most prominently the work of Bill Lands—indicates that an excessively high dietary intake of the *n*-6 PUFA linoleic acid (LA) increases the endogenous formation of *n*-6 HUFAs and their lipid mediators, while tending to suppress the production of health-promoting *n*-3 HUFAs and their derivatives. These findings provide both mechanistic plausibility and a clear biochemical prediction that shifts in HUFA balance may contribute to pathophysiological processes across multiple organs and systems. Consistent with Lands’ hypotheses, tissue HUFA profiles containing more than 50% *n*-6 HUFAs are associated with less favorable long-term health outcomes than profiles below this threshold [30,33,159]. Yet the extent to which these mechanistic and biochemical expectations have achieved empirical confirmation or clinical demonstration remains contested. As a result, the divergent viewpoints outlined here persist and, as recently emphasized, call for more definitive clarification [160].

### 3.5. The Availability of n-3 PUFAs in Individuals Consuming the “Western Diet” High in n-6 PUFAs Is Steadily Declining

The percentage of *n*-3 in HUFAs assessed in tissue, together with the omega-3 index (derived from the direct assessment of erythrocyte EPA + DHA as a percentage of total fatty acids), declined over the 20th century in the USA and likely elsewhere. The estimated percentage of *n*-3 in HUFAs diminished from about 37% in 1909 to about 23% in 1999, and the estimated average omega-3 index declined from 8.3 to less than 4 [94]. From these data, it is concluded that the net effect of increasing dietary LA is one likely important cause of a decreasing EPA and DHA status of human tissues over the 20th century. The reasons for this include the following:

- An impairment of the conversion of ALA to stearidonic acid (18:4*n*-3) and on to EPA (20:5*n*-3) due to competition of LA with ALA for D6-desaturase [161].
- An impairment of the conversion of EPA to DHA because of competition between *n*-6 and *n*-3 intermediates for the active site of D6-deasturase being dominated by the *n*-6 metabolites [162,163].
- An impairment of the incorporation of EPA, DPA (22:5*n*-3), and DHA into cell membranes due to competition with ARA (which is abundant) for esterification into the s*n*-2 position of phospholipids [93].

On the other hand, evidence supports the assumption that a reduction in LA intake enables ALA to compete more effectively for access to the active site of D6-desaturase so promoting EPA synthesis [16,164].

These observations clearly support Lands’ hypothesis that PUFAs and HUFAs of the *n*-6 and *n*-3 families interact metabolically, and that excessive availability of the *n*-6 PUFA substrate LA impairs the endogenous formation of *n*-3 HUFAs. This interpretation is further reinforced by the findings of Harnack et al. [165], who demonstrated a strong influence of the dietary *n*-6/*n*-3 PUFA ratio on the conversion of precursor PUFAs to HUFAs, underscoring not only the importance of adequate fatty acid intake but also of maintaining an appropriate ratio of LA to ALA. Another rare empirical demonstration of these competitive dynamics comes from the randomized controlled trial by Ramsden et al. [166], which simultaneously lowered dietary linoleic acid and increased *n*-3 EPA+DHA intake in patients with chronic migraine. Participants entered the study consuming a typical Western diet characterized by high linoleic acid intake and low *n*-3 HUFA status. The dietary interventions produced marked shifts in circulating and membrane HUFA profiles: lowering dietary LA reduced the *n*-6 HUFA percentage and increased *n*-3 HUFA levels even without additional *n*-3 intake, while the combined LA-lowering and *n*-3-increasing intervention produced the largest changes in HUFA balance and the strongest increases in EPA- and DHA-derived specialized pro-resolving mediators. These findings provide empirical support for the prediction that high background *n*-6 intake limits the incorporation of *n*-3 HUFAs, and that coordinated dietary modification can partially reverse this constraint.

### 3.6. The Appropriate Dietary n-3 HUFA Uptake Depends on the Individual Cellular n-6 HUFA Availability

A study of worldwide diversity in disease burdens and intakes of PUFAs led to estimates of how much added dietary *n*-3 HUFAs would be required for different populations to achieve a target health risk assessment (HRA) status of ≤50% *n*-6 in HUFAs [167]. Because of the large global variation in LA intake, that “HRA goal” might require added intakes of 1%E *n*-3 HUFAs for the USA, 0.5%E for Italy, 0.26%E for Denmark, and only 0.06%E for the Philippines [167]. For current US dietary patterns, it was estimated that a healthy dietary allowance for *n*-3 HUFAs would be 3.5 g/d for a daily caloric intake of 2000 kcal [167]. However, this daily amount of *n*-3 HUFAs would be extremely difficult to obtain from food alone, and providing it through oral supplements appears unrealistic for the general population. Moreover, natural *n*-3 HUFA resources would be rapidly depleted.

Fortunately, Hibbeln et al. also demonstrated that the required availability of *n*-3 HUFAs can likely be lowered to one-tenth of the amount otherwise needed by reducing dietary intake of *n*-6 PUFA-rich fats [167]. In practical terms, this means that—rather than increasing *n*-3 HUFA intake to 3.5 g/d to achieve an HRA status of ≤50% *n*-6 in HUFAs—the same target could be reached by lowering LA intake from its current US average of ~16.5 g/d (6.8%E) to ~2.5 g/d (1%E). Thus, Hibbeln’s analysis implies that the *n*-3 HUFA requirement is not fixed, but depends strongly on the competing availability of dietary *n*-6 PUFA substrates.

Interestingly, preliminary evidence was already provided by Wood et al. [168], who showed that a low-*n*-6 PUFA diet increases *n*-3 HUFA status in plasma phospholipids in humans. The authors concluded that reducing dietary LA intake to 2%E in a free-living population is feasible. Their intervention demonstrated that a low-LA diet reduces LA and total *n*-6 HUFA content in plasma and erythrocyte phospholipids, while simultaneously increasing *n*-3 HUFA content without any increase in dietary *n*-3 PUFA intake—presumably due to enhanced metabolic conversion of available *n*-3 precursors. Similar findings were reported by Chan et al. [17], where reduced LA intake resulted in higher EPA levels in plasma and cell lipids.

Thus, short-term reductions in dietary LA intake can improve *n*-3 HUFA status even without increasing dietary *n*-3 PUFA intake. The improvement would likely be even greater with higher dietary ALA intake and/or longer intervention periods.

## 4. What Data and Concepts May Disprove Lands’ Hypotheses?

Any disconfirming evidence would need to demonstrate that Lands’ hypotheses fail under controlled experimental conditions. In practice, however, such contradictions have not emerged. Instead, existing studies reveal quantitative limits, regulatory saturation, and physiological buffering—factors that refine but do not overturn Lands’ core logic. Thus, the question is not whether his hypotheses have been experimentally falsified—they have not—but rather what kinds of data could, in principle, challenge them. At this point, the focus necessarily shifts from mechanistic experiments to clinical studies, which operate under fundamentally different constraints and therefore cannot be expected to directly falsify mechanistic hypotheses.

The present contribution does not aim to provide a systematic review of all available clinical studies, nor would such an approach be appropriate for the purpose at hand. This is a narrative synthesis intended to summarize Lands’ central hypotheses and to re-contextualize their relevance. Moreover, a formal systematic review or meta-analysis would risk misleading conclusions if the included studies do not meet the methodological prerequisites implied by Lands’ framework—particularly the need to measure both PUFA families, to account for competitive dynamics, and to model their non-linear interactions. The selected clinical studies therefore serve only as illustrative examples, and some of them present with these broader limitations.

In recent years, a growing number of intervention studies have addressed questions that are broadly related to Lands’ hypotheses, including trials on DHA supplementation and neurodevelopment in preterm or term infants, omega-3 fatty acids and the risk of preterm birth, dietary modification of linoleic acid and omega-3 intake in migraine, and EPA/DHA supplementation in depressive disorders. A large meta analysis on omega-3 fatty acids and preterm birth reported reductions in early delivery risk, although these effects were achieved without systematic control of background linoleic acid intake or explicit targeting of *n*-3 HUFA status [169]. Likewise, the most recent Cochrane review on omega-3 fatty acids for depression in adults concluded that the overall certainty of evidence is low to very low, with small and clinically uncertain effects and substantial heterogeneity across trials [170]. These findings illustrate that, while clinically relevant, such studies do not constitute rigorous tests of Lands’ mechanistic framework. Most of them only test fragments of his hypotheses rather than their full mechanistic scope, and many identify methodological limitations—such as unrecognized confounders—only in retrospect. Yaqoob et al. likewise reported EPA/DHA incorporation without functional effects, possibly influenced by the high α-tocopherol content of the capsules used as supplements [171]. Moreover, many large trials and meta analyses on DHA supplementation in preterm or term infants, or on omega-3 fatty acids in pregnancy and neurodevelopment, have typically focused on increasing DHA intake against a background of largely uncontrolled linoleic acid consumption and without explicit targeting of *n*-3 HUFA status [172,173]. As a result, they provide important clinical information but only a partial and indirect test of Lands’ mechanistic hypotheses. The study by Ramsden and colleagues in patients with migraine probably comes closest to the criteria to be demanded by Lands. This randomized trial manipulated both dietary *n*-3 EPA+DHA and *n*-6 linoleic acid and showed that a combined increase in *n*-3 and reduction in linoleic acid produced the most favorable changes in headache outcomes and lipid mediators, which is qualitatively consistent with Lands’ predictions [166]. Yet, even in this carefully designed study, HUFA status was not the primary target variable, the intervention period was relatively short, and the findings are restricted to a single clinical condition.

From the perspective of Lands’ framework, more definitive tests would require interventions that systematically manipulate both *n*-6 linoleic acid and *n*-3 intake, explicitly aim at distinct HUFA status targets, and relate these to clearly defined, mechanistically plausible clinical endpoints. In this sense, many recent intervention studies have been “broadly testing” Lands’ ideas, but their designs have not yet been optimized to rigorously corroborate or falsify his hypotheses.

In their narrative review, Czernichow et al. evaluated the potential risks of dietary *n*-6 PUFAs for cardiovascular health [151]. Their aim was to assess evidence linking *n*-6 PUFA intake to CVD outcomes. Based on epidemiological data, they inferred that higher dietary *n*-6 PUFA intake significantly lowers LDL-cholesterol and several other CVD risk factors, including blood pressure, inflammatory markers, and obesity. Moreover, in their view, findings from prospective cohort studies and interventional trials converge toward a protective role of dietary *n*-6 PUFAs—particularly LA—against CVD. Consequently, the authors justify recommending a liberal intake of *n*-6 PUFAs, exceeding 5% and ideally approaching 10% of total energy intake. A more recent review by Maki et al. applied a similar evaluation approach and likewise concluded that the available evidence supports current recommendations to emphasize the consumption of *n*-6 PUFAs (especially LA) as a replacement for SFAs [174].

Recent cohort studies also point in the same direction. In particular, the FORCE consortium conducts meta-analyses of cohort-level data examining associations between blood or tissue fatty acid levels and various disease outcomes. The relationships between CHD risk and *n*-6 PUFAs were evaluated by Marklund et al. as part of a FORCE consortium analysis [45]. Total CHD events were inversely associated with LA (a 6% lower risk per interquartile range (IQ5R), i.e., comparing approximately the 10th to the 90th percentile of LA levels), whereas ARA levels showed no association with risk. For stroke, LA was associated with a 12% lower risk per IQ5R, again with no relationship observed for ARA [45]. Similarly, the risk of incident type-2 diabetes was 35% lower per IQ5R for LA, while ARA levels were unrelated to risk [175].

A similarly positive scenario—or at least no signal of harm—is found in evaluations of *n*-6 PUFAs in patients with non-alcoholic fatty liver disease (NAFLD) [176,177,178,179]. Comparably positive findings—or at least no concerning evidence—have been reported for patients with type 2 diabetes or elevated fasting glucose [180,181]. Not least, the questions raised by Lands regarding the pathogenetic significance of excessive dietary *n*-6/*n*-3 PUFA ratios, the potential dangers of excessive *n*-6 PUFA intake, the need for an upper toxicity limit, and the metabolic interactions between the two PUFA families continue to be contradicted in review articles up to the present day [47,48,182,183,184].

However, the broader evidence base underlying these LA-positive conclusions—across narrative reviews, prospective cohorts, pooled analyses, and meta-analytic consortia—shares several methodological limitations that constrain interpretability. Most studies did not assess baseline PUFA status and measured only LA as the PUFA endpoint, without parallel monitoring of endogenous *n*-3 PUFA or HUFA availability. This omission obscures the competitive interactions between the PUFA families that Bill Lands consistently emphasized. His framework describes *n*-3 and *n*-6 fatty acids as substrates competing for shared enzymatic pathways, formalized in his “competitive, hyperbolic equation”. These competitive dynamics determine HUFA balance, modulate the intensity of eicosanoid formation, and influence the likelihood of downstream “cascade overreactions”. Because these interactions are inherently non-linear, statistical models that treat PUFA exposures as independent, linear predictors risk generating results that are not physiologically valid. Moreover, fatty acid levels were typically obtained at a single time point, providing only a snapshot rather than a dynamic representation of HUFA balance over time. In addition, dietary substitution patterns (e.g., LA replacing saturated fat) cannot be disentangled from the biochemical effects of LA itself. Not least, residual confounding remains an inherent limitation of observational datasets—that is, unmeasured or imperfectly measured lifestyle and dietary factors may continue to influence the observed associations even after statistical adjustment. And finally, it is also important to note that circulating LA reflects recent dietary intake and substitution patterns rather than membrane HUFA composition; as such, inverse associations with circulating LA cannot be interpreted as evidence against the competitive HUFA dynamics central to Lands’ framework.

It should also be noted that most interventional studies examining the effects of LA were conducted over only weeks or months [185]. In contrast, potential detrimental effects of excessive *n*-6 PUFA intake may require years of exposure to become apparent. Interestingly, the formation of *n*-6-HUFA-derived eicosanoids can be reduced when large amounts of LA-rich vegetable oils are consumed [186]. This may reflect inhibition of LA conversion to ARA at very high LA intakes, as discussed by Lands [159]. Thus, high dietary LA may displace ARA in tissues, reducing the substrate available for synthesis of potentially harmful eicosanoids. However, high LA intake also impairs the formation of *n*-3 HUFAs and may displace EPA and DHA. Thus, net effects may be non-linear and difficult to predict. These actions should not be ignored when considering the impact of high LA intake. It is also important to consider how long such compensatory mechanisms persist and whether high LA intake may exert damaging long-term effects—questions that remain insufficiently investigated.

There are, however, data from human trials that at least suggest the need for caution regarding high intakes of LA. These include findings from “old” RCTs showing that men consuming higher amounts of seed-oil-based margarines had higher mortality than those consuming solid fats [187,188], although these results are difficult to interpret because industrial margarines of that era contained substantial amounts of trans fatty acids. More recently, evidence supporting increased caution toward an oversupply of *n*-6 PUFAs—at least relative to *n*-3 PUFAs—comes from a study by Zhang et al. [189] using the UK Biobank cohort. In this study, both *n*-3 and *n*-6 PUFAs in plasma were inversely associated with all-cause, cancer, and CVD mortality, but the associations were stronger for *n*-3 PUFAs. As a result, a strong positive association between the circulating *n*-6/*n*-3 PUFA ratio and the risk of all-cause, cancer, and CVD mortality was observed. However, broader discussions of the *n*-6/*n*-3 ratio debate and the apparent mechanistic–epidemiological discrepancies extend beyond the scope of this review but do not conflict with the mechanistic principles central to Lands’ framework.

In conclusion, the potential risks associated with dietary *n*-6 PUFA overload cannot be answered conclusively at present. This is underscored by conflicting evidence on associations between LA intake and various chronic diseases, as summarized by Mercola et al. [46]. However, these incongruent findings do not necessarily contradict Lands’ hypotheses. The study by Zhang et al. discussed above may offer an important new perspective on the contradictory evidence [189]. Moreover, it is not suggested that *n*-6 PUFA intake should be completely or invariably avoided—LA is essential and must be consumed. It is likely that a U-shaped curve exists, with an optimal range flanked by unfavorable outcomes at both very low and very high LA availability. Short-term high-dose LA intake may even have transiently beneficial effects by inducing substrate inhibition of desaturases, thereby slowing conversion to ARA. The concern, however, relates to the chronic and progressively increasing intake of LA, which may contribute to chronic disease. In individuals with demonstrated *n*-6 PUFA excess (elevated *n*-6 HUFA score), further increasing LA intake over the long term appears inappropriate and potentially detrimental. Until Lands’ hypotheses can be more definitively substantiated or refuted—depending on one’s interpretation of the available evidence—they warrant continued investigation, while both the public and patients should be protected from potential risks.

## 5. Strengths and Limitations

This article offers a conceptual and mechanistic synthesis of Lands’ hypotheses, integrating biochemical principles, quantitative modeling, and selected clinical evidence to clarify the relevance of HUFA-based metrics for contemporary nutritional science. A key strength of this contribution is its explicit focus on the competitive dynamics between *n*-6 and *n*-3 PUFA families, which provides a coherent framework for interpreting diverse findings across mechanistic studies, observational research, and dietary intervention trials. By highlighting the quantitative logic underlying HUFA balance, the article helps to reconcile seemingly contradictory results in the literature and identifies conditions under which dietary manipulations are most likely to influence tissue HUFA composition and downstream lipid mediator formation. Another strength is the integration of recent clinical studies—including those that simultaneously modify dietary linoleic acid and *n*-3 intake—into a mechanistic context that clarifies why some interventions yield stronger biochemical and clinical effects than others.

A further strength of this article lies in its effort to distill and articulate Lands’ extensive and often dispersed body of work into a set of coherent theses. By reassembling these mechanistic insights and placing them back into the contemporary scientific discourse, the article helps restore conceptual clarity to an area where key ideas have at times been overlooked, marginalized, or prematurely dismissed. This synthesis not only renews the visibility of Lands’ contributions but also reframes apparent discrepancies between basic biochemical research and clinical findings—not as contradictions to be dismissed, but as opportunities for renewed inquiry and more rigorous hypothesis testing. This perspective encourages a constructive interpretation of unresolved questions and highlights the potential for future studies to bridge existing gaps.

At the same time, several limitations should be acknowledged. This article is a narrative synthesis rather than a systematic review, and it does not aim to exhaustively summarize all available clinical or epidemiological studies. The selection of studies is therefore illustrative and focused on those most relevant to Lands’ mechanistic framework. Because many published trials do not measure HUFA status, do not manipulate both PUFA families simultaneously, or do not align their endpoints with mechanistic predictions, the available evidence base is inherently constrained. Furthermore, the article emphasizes biochemical and quantitative principles, which may not fully capture the complexity of long-term dietary patterns, lifestyle factors, or interindividual variability in PUFA metabolism. Finally, while the mechanistic logic of Lands’ framework is internally consistent and supported by several lines of evidence, definitive falsification or corroboration would require intervention studies specifically designed to manipulate both *n*-6 and *n*-3 intake with predefined HUFA targets—studies that are still scarce.

Taken together, these strengths and limitations reflect the intended scope of the article: to clarify the mechanistic foundations of Lands’ hypotheses, to contextualize existing evidence, and to identify the types of data and study designs that would be required to more rigorously test his framework in the future.

## 6. Summary and Concluding Remarks

Looking at Lands’ hypotheses and the evidence supporting them, the following picture emerges. PUFAs, HUFAs, and their derived lipid mediators may be considered pharmacologically active substances provided by nutrition. Their direct influence on gene expression may even be more significant than that of their derivatives. The decisive factor appears to be that *n*-6 and *n*-3 HUFAs metabolically affect each other and, even more importantly, differ in their efficacy to act, be it through their direct or mediator action. The dietary PUFA mixture may determine cellular fatty acid profiles and lipid mediator synthesis, giving rise to different organ- or system-functions and thus different non-energetic PUFA actions.

Against this background, it appears important to recognize that the oral intake of LA via seed oils (directly or indirectly via other foods) has risen in recent decades, and a resulting increase in *n*-6 PUFAs or HUFAs above desired levels in large parts of the population may reasonably be assumed. This underlines the need to define upper toxicity levels as well as a target availability of *n*-6 PUFAs, although no agreement has yet been reached on their determination. By contrast, the availability of *n*-3 HUFAs in individuals consuming the “Western diet” high in *n*-6 PUFAs appears to be steadily declining. This seems to be caused not only by a reduced uptake of *n*-3 PUFAs but also by a negative impact of *n*-6 PUFAs on *n*-3 PUFA metabolism. A variety of non-communicable diseases—including CVD—appear to be driven by an increased *n*-6 HUFA balance, although we are only just beginning to understand the underlying pathophysiology. Beyond that, the existing oversupply of *n*-6 PUFAs in the population may be an overlooked cause for conflicting results in RCTs investigating the clinical efficacy of dietary *n*-3 PUFAs.

Dietary interventions appear suitable to reduce the percentage of *n*-6 in HUFAs in tissues, to increase the endogenous availability of *n*-3 PUFAs, and to improve dysbalance-related pathomechanisms with consecutive health benefits and cost savings. However, a further important finding is that the appropriate dietary *n*-3 HUFA uptake appears to depend on the individual’s pre-existing cellular *n*-6 HUFA availability. High endogenous *n*-6 PUFA loads may require a high *n*-3 PUFA intake with the diet. Vice versa, lower endogenous *n*-6 PUFA availabilities may require a correspondingly lower *n*-3 PUFA intake. The same appears to apply to the therapeutic window of *n*-6 PUFAs—a high *n*-6/*n*-3 PUFA ratio may lower this window, while a physiological ratio may significantly increase it. Thus, combining a lower *n*-6 with higher *n*-3 PUFA intake appears to normalize an elevated *n*-6/*n*-3 PUFA ratio most effectively due to the predictable quantitative dynamics of the competing HUFA families. This inference may be of great importance in successfully implementing nutritional strategies aiming at normalizing an increased *n*-6/*n*-3 PUFA ratio, because neither the resources nor the population’s compliance are likely to be sufficient to balance high *n*-6 PUFA loads with high *n*-3 PUFA intakes.

Based on the conclusions outlined, measures to optimize *n*-6 and *n*-3 HUFA balances may be considered health-relevant and valid biomarkers for designing, as well as monitoring, successful nutrition strategies. In particular, the individual *n*-6 HUFA balance appears to be a valuable health risk assessment (HRA) measure because of its relationship to dietary nutrients and to pathophysiological outcomes. Achieving lower *n*-6 in HUFA balances in the range of 50% may help to improve health status, to reduce annual healthcare claim costs, and to ensure a return on investment in corresponding campaigns. Beyond that, new insights into the genetic variation in the enzymes involved in the metabolism of PUFAs may open up new perspectives. In the future, we may not only have to measure the fatty acid profiles in patients as well as in the population, but we may also need to assess their genetic profiles in order to identify high-risk patients in particular.

This discussion favors monitoring and limiting the *n*-6 PUFA supply. However, there are also data and concepts that may disprove all the discussed inferences. Therefore, more research is certainly needed on the biological interrelationships discussed and on strategies for averting (and reversing) this situation.

So, is Lands actually right? Unfortunately, we still cannot answer this question. If, however, Lands’ theses can be further corroborated—and the chances of that happening are probably not so small—then the healthcare implications would be substantial. It could mean that we have identified a major contributor to many of the ever-increasing non-communicable diseases. This would open a path toward reducing the incidence of cardiovascular diseases, type II diabetes, or cancers—morbidities that might be mitigated through causal prevention, thereby reducing the need for complex, costly, and profitable treatment procedures.

Bill Lands’ hypotheses therefore leave us with a significant need for action, one that currently appears undervalued. The central aim of this article is to revitalize and sustain interest in addressing this need. Hypotheses of this magnitude should be rigorously corroborated or falsified, but under no circumstances ignored.

Ultimately, the tension between robust biochemical logic and inconsistent epidemiological signals should not be interpreted as a dead end, but as an invitation. The hypotheses first articulated by Lands remain mechanistically compelling while still empirically unsettled. Addressing them with modern methodological rigor offers a clear path toward resolving long-standing ambiguities in the field.

## Figures and Tables

**Table 1:** Summary of Bill Lands’ key hypotheses regarding dietary polyunsaturated fatty acids (PUFAs), highly unsaturated fatty acids (HUFAs), metabolic competition, biomarker logic, and implications for chronic disease and public-health strategies.

No.	Hypothesis
**1**	The dietary mixture of polyunsaturated fatty acids (PUFAs) determines cellular fatty-acid profiles and thereby shapes the non-energetic biological actions of these lipids.
**2**	*n*-6 and *n*-3 highly unsaturated fatty acids (HUFAs) influence each other metabolically, differ in their biochemical efficacy, and give rise to distinct organ- and system-level functions.
**3**	The competition of *n*-6 and *n*-3 HUFAs for shared metabolic enzymes (COX, LOX, CYP) is the primary determinant of downstream lipid mediator profiles.
**4**	The quantitative relationship between dietary PUFA intake and HUFA composition is predictable and can be modelled with high accuracy, enabling mechanistic forecasting of biological outcomes.
**5**	The long-standing neglect of dietary PUFA imbalance may contribute to the continued rise in non-communicable diseases.
**6**	Dietary interventions can lower the percentage of *n*-6 in HUFA, with potential health benefits and associated reductions in healthcare costs.
**7**	The individual *n*-6 HUFA profile serves as a valuable surrogate biomarker because it reflects both dietary inputs and pathophysiological outcomes.
**8**	Combining reduced *n*-6 with increased *n*-3 PUFA intake most effectively lowers the percentage of *n*-6 in HUFA, owing to the predictable quantitative dynamics of the competing HUFA families.
**9**	Failure to account for the population-wide oversupply of *n*-6 PUFAs may help explain inconsistent results in randomized controlled trials evaluating the clinical efficacy of *n*-3 PUFAs.
**10**	Measures of basal as well as final *n*-6 and *n*-3 HUFA status should be considered important and valid biomarkers for designing and monitoring effective nutritional strategies.
**11**	A range of non-communicable diseases appears to be associated with elevated *n*-6 HUFA levels, and the underlying pathophysiological mechanisms are increasingly understood.
**12**	In cardiovascular disease, preliminary evidence already suggests a potential causal role for an increased *n*-6 HUFA profile.
**13**	Achieving an *n*-6 HUFA percentage near 50% may help reduce annual healthcare expenditures and improve the cost-effectiveness of public-health interventions.

**Table 2:** Evidence-based concepts supporting Lands’ hypotheses.

No.	Concept
**1**	The *n*-6/*n*-3 HUFA balance governs inflammatory, immunologic, and metabolic signaling
**2**	Excessive *n*-6 PUFA and HUFA abundance drives molecular, cellular, and organ-level pathomechanisms linked to chronic disease
**3**	Increasing linoleic acid intake may amplify HUFA-mediated pathomechanisms in *n*-6-dominant physiological states
**4**	The concept of a dietary toxicity threshold for linoleic acid appears to be supported by available evidence, yet remains debated
**5**	The availability of *n*-3 PUFAs in individuals consuming the “Western diet” high in *n*-6 PUFAs is steadily declining.
**6**	The appropriate dietary *n*-3 HUFA uptake depends on the individual cellular *n*-6 HUFA availability.

**Table 3:** Molecular, cellular and organ systems regulated by PUFAs, HUFAs and their oxylipin derivatives.

Systems	Examples
Molecular: Cell signalling, gene expression and protein production Ion channels and membrane transporters	Probably all cell types [6,96] Many cell types including cardiomyocytes [97,98] and neurones [99]
Cellular: Activation, proliferation and responsiveness Mitochondrial biogenesis and function	Probably all cell types, including intestinal epithelial cells [100,101], skin cells [100,102], immune and inflammatory cells [71,103], platelets [6,104] and endothelial cells [105,106] Probably all cell types [107,108]
Organ development:	Probably all organs, but especially eye and brain [109,110,111,112]
Organ function and (life-stage) physiology:	
Fertility (both male and female)	[113,114,115]
Pregnancy	[116,117]
Parturition	[118,119]
Vision	[120]
Brain function/cognition	[111,112,121]
Cardiac function	[122,123]
Liver function	[124,125]
Renal function	[126,127]
Skeletal muscle function	[128,129]
Bone homeostasis	[130,131]
Inflammation	[67,71]
Immune defence	[103]
Haemostasis	[6,104,132]
Vasoconstriction/vasodilation/blood flow/blood pressure	[133,134]
Wound healing	[135,136]
Lipid metabolism (synthesis, oxidation, deposition, mobilisation)	[83,137]

**Table 4:** Morbidities that may result from excessively high abundance of *n*-6 PUFAs and HUFAs.

Impact of Excessive High Abundance of *n*-6 PUFAs and HUFAs	Expected Morbidity *
Low *n*-3 HUFA availability in early life	Poorer visual development
Low *n*-3 HUFA availability in early life	Poorer cognitive development (childhood learning and behavioural disorders)
Low *n*-3 HUFA availability and excessive pro-parturition *n*-6 HUFA-derived oxylipins during pregnancy	Pre-term birth
Low *n*-3 HUFA availability during pregnancy and excessive *n*-6 HUFA-derived oxylipins	Gestational diabetes
Low *n*-3 HUFA availability during pregnancy and excessive *n*-6 HUFA-derived oxylipins	Post-natal depression
Low *n*-3 HUFA availability and excessive pro-proliferative, anti-apoptotic *n*-6 HUFA-derived oxylipins	Many cancers
Low *n*-3 HUFA availability and excessive pro-inflammatory *n*-6 HUFA-derived oxylipins	High-grade inflammatory conditions (rheumatoid arthritis, inflammatory bowel diseases, inflammatory skin diseases)
Low *n*-3 HUFA availability and excessive pro-inflammatory *n*-6 HUFA-derived oxylipins	Migraine, pain
Low *n*-3 HUFA availability and excessive pro-allergic *n*-6 HUFA-derived oxylipins	Allergy, asthma
Low *n*-3 HUFA availability and excessive pro-inflammatory *n*-6 HUFA-derived oxylipins	Low-grade inflammatory conditions (cardiovascular diseases (e.g., coronary heart disease, peripheral vascular disease, stroke), metabolic diseases (e.g., type-2 diabetes, fatty liver disease, more severe fatty liver disease), kidney disease, cognitive decline, loss of lean mass (muscle and bone → sarcopenia)
Low *n*-3 HUFA availability and excessive pro-inflammatory *n*-6 HUFA-derived oxylipins	Psychological and psychiatric diseases
Low *n*-3 HUFA availability and excessive pro-inflammatory *n*-6 HUFA-derived	Poor wound healing
Low *n*-3 HUFA availability and excessive pro-inflammatory *n*-6 HUFA-derived oxylipins	Critical illness following a severe physical insult

* *n*-3 HUFAs (EPA and DHA) have been shown to lower the risk of each of these morbidities.

**Table 5:** Overview of Dietary Linoleic Acid (LA) Intake Recommendations from Major Institutions and Selected Experts.

Institution/Source	LA Recommendation	Approx. % of Energy	Notes/Basis
**DGE** **(D-A-CH Reference Values, 2020)** [143]	2.5% of energy as LA + 0.5% as ALA (minimum to prevent deficiency)	~2.5%	Essential minimum; not defined as optimal intake
**EFSA** **(Europe, 2010)** [155]	Adequate intake: 10 g LA per day for adults	~4%	No upper limit defined
**UK SACN** **(UK 2025)** [158]	Up to 10% of energy as *n*-6 PUFAs (mainly LA) and not less than 1% of energy	~1–6% (typical range), proportion of the population consuming *n*-6 PUFAs in excess of about 10 EN% should not increase	Specifications in % of energy excluding ethanol; no explicit optimal target
**UK COMA** **(UK, 1991)** [156,157]	Caution above 6% of energy; >10% not recommended;	–	1991: Safety of >6% considered insufficiently tested. 1994: Explicit recommendationthat population intakes should not exceed 10% of energy
**AHA** **(USA, Guideline, 2023)** [154]	Higher PUFA intake encouraged; no explicit LA upper limit	–	Focus on replacing saturated fat with MUFA/PUFA; no LA/ALA ratio addressed
**AHA** **(USA, 2009)** [150]	5–10% of energy as *n*-6 PUFAs (mainly LA)	~5–10%	Suggested CHD risk reduction; higher intakes considered safe
**Holman** **(EFA deficiency research, 1964)** [149]	1.4% of energy as LA sufficient to prevent deficiency	~1.4%	Based on infant studies; foundational for EFA requirements
**Lands** **(2008)** [30]	Recommended intake ≈ 0.5%; upper safe limit ≈ 2%	0.5–2%	HUFA-based evaluation: minimal requirement, low upper safe limit

Legend: This table is based on established dietary fat recommendations and key scientific publications.

## Data Availability

No new data were created or analyzed in this study.
